# Modelling the effects of human SUR1 R1420H variation on insulin secretory function using isogenic iPSC-derived pancreatic islets

**DOI:** 10.1007/s00125-025-06605-1

**Published:** 2025-11-25

**Authors:** Anup K. Nair, Katiya Barkho, Koushik Ponnanna Cheranda, Michael Traurig, Jeffrey R. Sutherland, Divya Anup, Clifton Bogardus, Leslie J. Baier

**Affiliations:** Phoenix Epidemiology and Clinical Research Branch, National Institute of Diabetes and Digestive and Kidney Diseases, National Institutes of Health, Phoenix, AZ USA

**Keywords:** *ABCC8*, Diabetes, Dorzagliatin, *G6PC2*, Hyperinsulinaemia, iPSC-derived pancreatic islets, iPSCs, Pancreatic islets, SUR1

## Abstract

**Aims/hypothesis:**

An R1420H variation in sulfonylurea receptor 1 (SUR1), a subunit of the K_ATP_ channel, was previously identified in an Indigenous community in Arizona where a homozygous carrier (1420HH) had hyperinsulinaemic hypoglycaemia during infancy (HHI), suggestive of a K_ATP_ channel loss of function (LoF). Interestingly, heterozygous carriers of this variation (1420RH, occurring in 3% of the community), had a twofold increased risk of type 2 diabetes. We aimed to create an isogenic induced pluripotent stem cell (iPSC)-derived pancreatic islet (SC-islet)-based platform to test whether the R1420H variant results in K_ATP_ channel LoF, and to examine the distinct temporal effects of SUR1 1420HH and 1420RH K_ATP_ channel variations on insulin secretion from developing and mature SC-islets.

**Methods:**

Using CRISPR-Cas9, isogenic iPSCs with all three genotypes (SUR1 1420RR, 1420RH and 1420HH) were generated from two different parental Indigenous American iPSC lines (IS1, isogenic cell lines derived from parental cell line 1; and IS2, isogenic cell lines derived from parental cell line 2). These isogenic cell lines were used to generate immature SC-islets (resembling fetal islets) and mature SC-islets (resembling adult islets), which were used to assess insulin secretion dynamics during different stages of development and identify differences in gene expression by single-cell RNA-seq. This study was consistent with the CONSIDER statement for research studies among Indigenous American communities.

**Results:**

Immature SUR1 1420HH SC-islets secreted 3.4-fold (IS1, *p*<0.001) and 4.2-fold (IS2, *p*=0.001) more insulin under basal conditions than normal (SUR1 1420RR) SC-islets. Modest hyperinsulinaemia was also seen in immature SUR1 1420RH SC-islets (2.2-fold [IS1] and 2.3-fold [IS2]) but the results were not statistically significant. After maturation, the 1420HH SC-islets failed to achieve glucose responsiveness whereas the 1420RH SC-islets achieved biphasic insulin secretion but had significantly lower glucose responsiveness than normal SC-islets (AUC for insulin secretion [as a % of total insulin] under high glucose challenge: 1.04 vs 0.56 in normal vs 1420RH SC-islets, *p*<0.001). Diazoxide reduced hyperinsulinaemia in SUR1 1420RH and 1420HH immature SC-islets, while tolbutamide elicited a greatly diminished or undetectable insulin secretory response from mature SUR1 1420RH SC-islets (13.2-fold increase in insulin secretion) and 1420HH SC-islets (1.9-fold increase) compared with normal SC-islets (31.5-fold increase). Results were directionally comparable for both IS1 and IS2 SC-islets. SUR1 1420RH SC-islets also responded to the glucokinase activator dorzagliatin with improvement in first-phase insulin secretory response (first-phase stimulation index: 3.9-fold vs 7.3-fold, *p*=0.01 [IS1, 11 mmol/l glucose ± dorzagliatin]; 5.5-fold vs 9.0-fold, *p*=0.13 [IS1, 20 mmol/l glucose ± dorzagliatin]). Single-cell RNA-seq identified dysregulated genes in SUR1 1420RH SC-beta cells, including lower expression of glycolytic genes and upregulation of *G6PC2*, which could explain the lower insulin secretory response to glucose.

**Conclusions/interpretation:**

A SUR1 R1420H variant identified in an Indigenous American population causes hyperinsulinaemia in homozygous immature SC-islets during basal conditions and these SC-islets fail to achieve glucose responsiveness after maturation. In the heterozygous state, modest hyperinsulinaemia is observed in immature SC-islets, which after maturation have significantly lower glucose responsiveness. These results demonstrate that SUR1 R1420H is a K_ATP_ channel LoF variation and suggest that a lower insulin secretory response during adulthood is the cause of the higher type 2 diabetes risk in individuals heterozygous for this variation. We also show that the isogenic iPSC-based platform can be used to test therapeutic agents to treat HHI in infants homozygous for LoF K_ATP_ channels and screen for drugs that can improve glucose-responsive insulin secretion in adult heterozygous carriers.

**Data availability:**

See the database of Genotypes and Phenotypes (dbGaP; dbgap.ncbi.nlm.nih.gov/home; accession no.: phs002490.v1.p1) for details concerning data requests. All source codes can be found in the GitHub repository under https://github.com/Koushik-Cheranda/SC-islet-scRNAseq-analysis-R-codes.

**Graphical Abstract:**

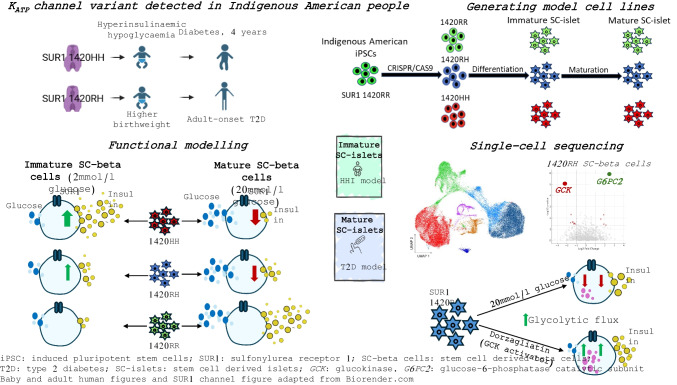

**Supplementary Information:**

The online version contains peer-reviewed but unedited supplementary material available at 10.1007/s00125-025-06605-1.


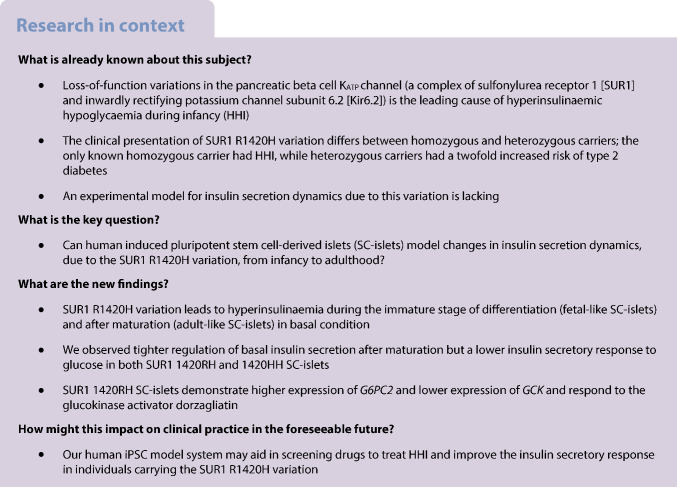


## Introduction

Monogenic causes of pancreatic beta cell dysfunction leading to hyperinsulinaemic hypoglycaemia during infancy (HHI) include gain-of-function variations in glucokinase (GCK) [1–3], which catalyses the first step in glycolysis, and loss-of-function (LoF) variations in either the inwardly rectifying potassium channel subunit 6.2 (Kir6.2, encoded by *KCNJ11*) or the sulfonylurea receptor 1 (SUR1*,* encoded by *ABCC8*), which together form the beta cell ATP-sensitive potassium channel (K_ATP_ channel) and couple ATP generation to insulin secretion [4–6]. Most cases of HHI are due to recessively inherited LoF Kir6.2 or SUR1 variations, leading to either the K_ATP_ channel not responding to raised intracellular ADP levels in low glucose conditions and remaining in a closed configuration, or the K_ATP_ channel being absent from the plasma membrane [7–10]. This results in a constant depolarisation of the membrane, leading to insulin hypersecretion and subsequent hypoglycaemia. Previously, we identified an R1420H variation (rs1272388614) in SUR1 in 3.3% of individuals from a community of Indigenous American people living in Arizona [11]. Heterozygous carriers of this variation had a 170 g increase in birthweight (consistent with fetal hyperinsulinaemia), a lower BMI during adulthood and a doubling of type 2 diabetes risk with a 7 year earlier onset. The only known homozygous carrier had HHI and was diagnosed with diabetes before the age of 4 years [11]; however, experimental evidence showing that this is a K_ATP_ channel LoF variation is lacking. Given the clear impact on type 2 diabetes among heterozygous carriers, distinct from typical forms of type 2 diabetes, it is critical to understand the molecular mechanism behind the switch from hypersecretion of insulin during in utero development and infancy to impairment in insulin secretion, which is a hallmark of type 2 diabetes, at later ages.

Human induced pluripotent stem cell (iPSC)-derived islets (SC-islets) have emerged as an attractive system to model the effect of pathogenic variants on pancreatic islet development and function. A study by Lithovius et al has previously used SC-islets to model loss of K_ATP_ channel function by using a known LoF variation (V187D) in SUR1 [12, 13]. The study demonstrated hypersecretion of insulin in basal condition in SC-islets with the LoF variation and identified a higher proportion of SC-beta cells and higher proliferation rate in SC-islets with the LoF K_ATP_ channel variation [13]. However, because of the lack of protocols to develop functional SC-islets with stimulus–secretion coupling at the time of study, their model did not recapitulate the effect of glucose stimulation on insulin secretion [13]. Moreover, the study only focused on the effect of the variation in the homozygous state [13]. More recently, protocols have been developed to generate SC-islets with stimulus–secretion coupling [14, 15]. In this study, we used an updated isogenic iPSC-derived pancreatic islet-based platform that generates SC-islets with stimulus–secretion coupling to test whether the SUR1 R1420H variation results in hyperinsulinaemia in basal condition. We used this platform to examine the temporal effects of the SUR1 R1420H variation on glucose-stimulated insulin secretion (GSIS), in both the homozygous and heterozygous state, in the developing pancreatic islets. We also examined the transcriptomic effects of early hyperinsulinaemia on SC-islets and modelled the later insulin secretory defect in the same system.

## Methods

### Ethics statement

The study was approved by the Institutional Review Board of the National Institute of Diabetes and Digestive and Kidney Diseases, NIH (protocol code 13-DK-N151). All participants provided informed consent. The study results, manuscript and informed consent forms were presented to and reviewed by the Indigenous Tribal Research and Review Committee (on 19 August 2024 and 18 November 2024) and the Indigenous Tribal Health and Social Standing Committee (on 24 September 2024 and 24 December 2024). The work adhered to the recommendations in the CONSIDER statement for reporting health research in Indigenous individuals [16]. A checklist detailing each point in the eight domains of the CONSIDER statement is shown as Appendix 1 in the electronic supplementary material (ESM).

### Human iPSC derivation and culture

Human iPSCs were derived from peripheral blood mononuclear cells (PBMCs) at the NIH-National Heart, Lung, and Blood Institute (NHLBI) iPSC core facility using a Sendai virus-based reprogramming kit (Thermo Fisher, Waltham, MA, USA). The PBMCs were obtained from two unrelated Indigenous American adult women (age >18 years) who were previously diagnosed with type 2 diabetes. These individuals provided informed consent for the generation of iPSCs. iPSC 1 (HT273A) was homozygous for the normal arginine allele (1420RR) and iPSC 2 (HT261B) was heterozygous (1420RH) for the SUR1 R1420H variation (NM_001351296.2:c.4259G > A). Tests for Sendai virus and mycoplasma (universal mycoplasma detection kit, catalogue no. 30-1012K; ATCC, Manassas, VA, USA) were routinely performed during passaging and the iPSCs were characterised for genomic stability, pluripotency and differentiation potential (ESM Fig. 1). The iPSCs were initially maintained in mTeSR1/Matrigel culture system (catalogue no. 85850; Stem Cell Technologies, Vancouver, BC, Canada / catalogue no. 354227; Corning, Corning, NY, USA) and then adapted to DEF-CS 500 culture system (catalogue no. Y30010; Takara Bio, San Jose, CA, USA). All downstream iPSC cultures were performed using DEF-CS 500 (Takara Bio) culture system. See ESM Methods for more details.

### Generation of isogenic iPSCs

**Fig. 1:**
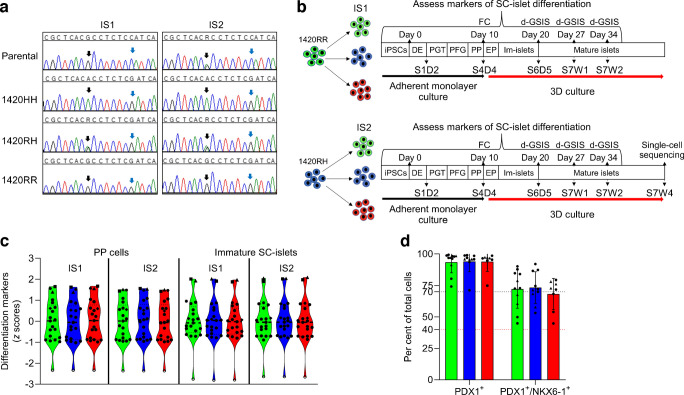
Generation and characterisation of isogenic iPSCs used for generating SC-islets (see also ESM Figs 1–3). (**a**) Chromatograms confirming the correct genotypes (black arrows) and protospacer adjacent motif site synonymous changes (blue arrows) in the parental and edited iPSCs. (**b**) Outline of the seven-stage, 34-day differentiation protocol used to generate SC-islets and the assays performed at each stage. For the detailed differentiation protocol see ESM Table 1. (**c**) Relative mRNA expression of selected stage-specific differentiation marker genes shown as *z* scores in PP cells and in immature SC-islets generated from IS1 and IS2 cell lines. The black dots represent genes shown in ESM Fig. 4 and ESM Fig. 5. Open circles, *OCT4*; triangles, *INS*; squares, *GCG*; diamonds, *NKX6-1*; hexagons, *PDX1*. Black dashed lines represent the median. For IS1 cells (differentiations 2–6): green, 1420RR (*n*=7); blue, 1420RH (*n*=6); red, 1420HH (*n*=4). For IS2 cells (differentiations 5–7): green, 1420RR; blue, 1420RH; red, 1420HH (all *n*=3). (**d**) Flow cytometry results of PP (S4D4) cells stained for PDX1 and NKX6-1. Circles, PP cells generated using the IS1 cell line; triangles, PP cells generated using the IS2 cell line. For IS1 cells (differentiations 1–6): green bars, 1420RR (*n*=8); blue bars, 1420RH (*n*=7); red bars, 1420HH (*n*=5). For IS2 cells (differentiations 5–7): green bars, 1420RR; blue bars, 1420RH; red bars, 1420HH (all *n*=3). Black dotted line, cut-off used for minimum per cent of PDX1^+^ cells to continue differentiation; red dotted line, cut-off used for minimum per cent of PDX1^+^/NKX6-1^+^ cells to continue differentiation. Data are presented as mean ± SD. D, day; EP, endocrine progenitor cells; IM-islets, immature SC-islets; PFG, posterior foregut cells; PGT, primitive gut tube cells; S, stage; W, week

Guide RNA (sgRNA) generation, iPSC editing and single-cell cloning was performed using the Cellartis iPSC rCas9 Electroporation and Single-Cell Cloning System (catalogue no. 632643; Takara Bio) following the manufacturer’s instructions. The 20 bp target sequence used for the generation of sgRNA was 5′-GACGGGGTCCTGCAGGATGA-3′. An asymmetrical donor DNA (ultramer DNA oligo; IDT, Coralville, IW, USA) was used to either introduce or correct the variation (ESM Fig. 2a). See ESM Methods for more details. Both parental iPSCs underwent CRISPR-cas9 editing to either introduce or to correct the variation, generating two sets of isogenic cell lines (isogenic cell lines 1 [IS1] and isogenic cell lines 2 [IS2]) with all three genotypes (SUR1 1420RR, 1420RH and 1420HH). The isogenic cell lines were sequenced (Fig. 1a) and karyotyped (ESM Fig. 2c, d) to confirm the edits, absence of CRISPR off-target effects (ESM Fig. 2b) and genomic stability.

### Genomic stability

Screening for genomic abnormalities was performed using G-band karyotyping at WiCell (Madison, WI, USA). Cells from the last passage used for cryopreservation were sent for karyotyping and all further experiments were carried out using this batch of cryopreserved cells. For karyotyping, 20 metaphase cells were counted, eight were analysed and four had karyograms to screen for chromosomal abnormalities.

### Off-target effect screening

The top 15 predicted off-target regions were identified using the CCTop-CRISPR/Cas9 target online predictor tool (https://cctop.cos.uni-heidelberg.de:8043/, accessed 16 May 2022). Sanger sequencing was used to compare the sequence at these regions between the isogenic cell lines and the parental cell line.

### Pluripotency and differentiation potential

Pluripotency and differentiation potential were assessed at the iPSC stage and after directed differentiation towards definitive endoderm (DE) by assessing stage-specific expression of markers by flow cytometry (TRA-1-60 and stage-specific embryonic antigen 4 [SSEA4] for pluripotency; C-X-C chemokine receptor type 4 [CXCR4] and CD117 for DE).

### Differentiation of isogenic iPSCs to SC-islets

Before differentiation to generate SC-islets, all isogenic iPSCs were assessed for pluripotency and the potential to generate DE. We used a cut-off of >90% cells co-expressing TRA-1-60 and SSEA4 in the undifferentiated stage and >80% cells co-expressing CXCR4 and CD117 after 2-days of directed differentiation towards DE (ESM Fig. 3a–d). Eighteen isogenic iPSCs (nine IS1 and nine IS2) that passed the above criteria were selected for further differentiation. For generating SC-islets, a seven-stage differentiation protocol modified from previous publications was used [14, 15, 17–20]. This protocol consistently led to the generation of SC-islets with a biphasic response to glucose by the end of stage 7, while stage 6 SC-islets are non-glucose responsive and resemble fetal islets. For differentiation, iPSCs were seeded at a density of 1.0×10^5^ cells/cm^2^ into tissue culture flasks coated with iMatrix-511 (0.5 µg/cm^2^; catalogue no. NP892-012; Stemgent, Cambridge, MA, USA). Differentiation was initiated after 48 h by rinsing cells with Dulbecco’s PBS (DPBS, Thermo Fisher) and replacing DEF-CS media with day 0 differentiation media. The medium was then changed every day until the end of differentiation. The differentiation media composition and complete protocol are shown in ESM Table 1. We performed a total of 13 independent differentiations using a combination of IS1 and IS2 iPSCs. Each differentiation included isogenic cells with different genotypes differentiated at the same time. All assays were performed at the same time. Isogenic cell lines and iPSCs used during each differentiation are shown in ESM Table 2.

### RNA isolation and quantitative real-time PCR

RNA was isolated using the RNeasy plus micro kit (catalogue no. 74034; Qiagen) and 1–2 µg of total RNA was converted to cDNA using the high-capacity RNA-to-cDNA kit (catalogue no. 4387406; Thermo Fisher). Real-time PCR was performed using validated gene-specific primers (listed in ESM Table 3) and the PowerUP SYBR green master mix (catalogue no. A25777; Thermo Fisher). Real-time PCR was primarily used to ensure proper differentiation by assessing the expression of stage-specific marker genes. *TBP* was used for normalisation and relative expression was analysed using the ${2}^{-\Delta \Delta {\text{C}}_{\text{t}}}$ method [21]. See ESM Methods for more details.

### Flow cytometry

Staining for flow cytometry detection of surface antigens (TRA-1-60, SSEA4, CXCR4 and CD117) and intracellular antigens (pancreatic and duodenal homeobox 1 [PDX1], NK6 homeobox 1 [NKX6-1], insulin [INS] and glucagon [GCG]) was performed as described previously [22]. Antibodies used for flow cytometry are shown in ESM Table 4. See ESM Methods for details.

### Dynamic GSIS

Dynamic GSIS (d-GSIS) was performed using the Peri5 system (Biorep, Miami, FL, USA), which was set up as per the manufacturer’s instructions. Approximately 100 immature SC-islets and 70 mature SC-islets were first perifused with KRB containing 2 mmol/l glucose for equilibration (90 min) followed by KRB containing different glucose concentrations and/or drugs (diazoxide [catalogue no. S4030, Selleckchem], tolbutamide [catalogue no. T0891, Millipore Sigma] and dorzagliatin [catalogue no. HY-109030, MedChem Express]) as indicated in the figure legends at a flowrate of 100 μl/min. The effluent was collected every minute and assessed for secreted insulin using human insulin ELISA kit (catalogue no. 10-1113-01; Mercodia, Winston Salem, NC, USA). SC-islets were collected from chambers at the end of the perifusion to quantify total insulin and total DNA (Quant-iT PicoGreen dsDNA Assay Kits; catalogue no. P7589; Thermo Fisher) for data normalisation (see ESM Methods for details). All perifusion results and exposure time reported take into account the ~2 min time delay from when the solution change happens to actual exposure.

### Immunocytochemistry

Staining for immunocytochemistry was performed as previously reported [22]. See ESM Methods for details.

#### Hormone content

Hormone content was measured as described previously [17]. See ESM Methods for details.

#### Single-cell RNA library preparation

SC-islets used for single-cell RNA-seq were from four independent differentiations using nine IS2 cell lines. Three cell lines, one for each genotype, were used in two independent differentiations. Single-cell libraries were prepared following the 10X Genomics Single Cell 3′ v3 Reagent Kit and protocol as per the manufacturer’s instructions (10X Genomics, Pleasanton, CA). Libraries were sequenced using an Illumina NovaSeq 6000 System at SeqMatic (Fremont, CA) at the recommended read setting: read 1, 26 bps; read 2, 91 bps; I7 index, 8 bps. See ESM Methods for details.

#### Single-cell RNA data processing and transcriptomic profiling

The raw data files (fastq) from each sample were processed using the 10X Cell Ranger Single Cell Software Suite (v5.0.1) and aligned to the human reference genome (GRCh38). The unique molecular identifier (UMI) matrices were filtered to remove empty droplets using DropletUtils (v1.22.0) [23]. Filtered UMI counts were exported to Seurat (v4.4.0) [24] using the function *CreateSeuratObject* (min.cells=3, min.features=200) for single-cell analysis in R studio (v2024.4.1.748), R (v4.4.2). Ambient RNA contamination was removed using SoupX (v1.6.2) [25]. A gene cut-off (>2500 and <8000), UMI cut-off (>5000) and mitochondrial fraction cut-off (<25%) were used to identify viable cells. Cells with no UMI counts for housekeeping genes *ACTB* and *GAPDH* were filtered out. DoubletFinder (v2.0.4) was used to identify and remove potential doublets [26]. The analysis was carried out using 20 principal components (PCs), an assumed doublet rate of 7.5% and a pK value corresponding to the maximum BCmetric obtained from the *find.pK* function. SCTransform [v0.4.1] [27], as implemented in Seurat, was used for normalisation and variance stabilisation of expression count matrices followed by initial assessment of clustering for each differentiation separately and without data integration. For each differentiation we identified cells/clusters expressing *INS*, *GCG* or both using the *FeaturePlot* function in Seurat and then proceeded with unsupervised cell clustering by integration of all 12 samples to mitigate bias. We first identified 3000 highly variable genes (HVGs) using the *SelectIntegrationFeatures* in each dataset for integration. Anchor genes were identified using *FindIntegrationAnchors* and integrated using *IntegrateData* by applying canonical correlation analysis. PCs for the HVGs were computed using *RunPCA* function and a nearest neighbour graph was constructed using *FindNeighbors* with the first 30 PCs. Cells were clustered using *FindClusters* with a resolution parameter set to 0.2. Finally, Uniform Manifold Approximation and Projection (UMAP) dimensional reduction was applied on the clustered cells using *RunUMAP* function. Visualisations were carried out using *VlnPlot*, *DimPlot*, *FeaturePlot* and *DoHeatmap* functions within the Seurat package and *VlnPlot _scCustom* function from scCustomise (v2.1.2) R package [28]. Cluster annotation/cell type identification was carried out based on differential gene expression analysis (negbinom: logFC ≥ 0.25; min.pct=0.1; Bonferroni-adjusted *p*<0.01). To remove polyhormonal cells from the iPSC-derived beta cell (SC-beta) and iPSC-derived alpha cell (SC-alpha) cluster, we analysed the barcodes present in the SC-beta and SC-alpha cell clusters to identify barcodes that had very high UMI counts for both *INS* and *GCG*. Any barcode with a log-normalised count of >5.4 for *INS* in the SC-alpha cluster and >4.5 for *GCG* in the SC-beta cluster were removed. *FindMarkers* (assay = ‘SCT’, slot = ‘counts’, test.use = ‘negbinom’) with or without ‘sample’ as the latent variable as implemented in Seurat was used for differential gene expression analysis.

#### Statistical analyses and data processing

PRISM 10 (v.10.2.2, Graph Pad, CA, USA) was used for statistical analyses and graphing the data. For analyses, data collected from different isogenic cell lines with the same genotype from independent differentiations (indicated in figure legend) were pooled together. All statistical comparisons were made between data pooled from normal SC-islets (1420RR) and either SC-islets with heterozygous K_ATP_ channel LoF variation (1420RH) or SC-islets with homozygous K_ATP_ channel LoF variation (1420HH). The *n* values indicated in the figure legends represent the number of samples/cell lines rather than the number of differentiations, which is indicated separately. All analyses were performed using an unpaired *t* test (two-tailed) unless otherwise indicated in the figure legends. Any correction for multiple comparison and statistical parameters is indicated in the figure legends. For calculating insulin secretion AUC under high glucose exposure, secretion in basal glucose for each genotype was used as the baseline to calculate the AUC in PRISM. The stimulation index (SI) was calculated as fold increase in insulin secretion over basal (2 mmol/l glucose stimulation). Basal insulin secretion was calculated as an average of insulin secretion every minute during basal conditions (minutes indicated in figure legends). First- and second-phase SIs were obtained by calculating the average SI during the first and second phases (minutes indicated in figure legends) for each experiment. Statistical analyses used for single-cell sequencing data are described above and in the results section. Researchers were not blinded to the genotype of the samples.

## Results

### SUR1 R1420H variation does not affect the generation of pancreatic progenitors

The human iPSC line HT273A was derived from an Indigenous American individual homozygous for the Arg (R; reference allele) at position 1420 of SUR1. We used CRISPR-cas9 to introduce the His-allele (H) at position 1420 (changing nucleotide G to A at NC_000011.9: g.17417205) and derived nine isogenic cell lines with all three genotypes (IS1 cell lines: 2 1420RR, 4 1420RH, 3 1420HH) by single-cell cloning (Fig. 1a). This ensured that all nine cell lines had undergone similar stress and culture condition during derivation. To further increase the reliability of our system, we used another iPSC line (HT261B) derived from an unrelated Indigenous American individual heterozygous for the variant (1420RH). Using CRISPR-Cas9 we derived a second set of nine isogenic cell lines with the three genotypes (Fig. 1a, IS2: 3 1420RR, 3 1420RH, 3 1420HH). These 18 cell lines were then used in different independent experiments to generate glucose-responsive SC-islets using a modified seven-stage differentiation protocol (Fig. 1b). For each differentiation, efficiency was monitored by real-time PCR analysis of islet developmental markers (Fig. 1c, ESM Figs. 4–6) and by flow cytometry analysis of pancreatic progenitor (PP) formation (stage 4 day 4 [S4D4]). Flow cytometry identified a median of 93.9% PDX1^+^ cells and 72.2% PDX1^+^ NKX6-1^+^ cells; the differentiation efficiency did not differ by genotype and was similar for both IS1 and IS2 cell lines (Fig. 1d). Consistent with previous reports that K_ATP_ channel variations do not affect PP generation [13], no significant increase in *ABCC8* or *KCNJ11* expression was observed relative to iPSCs (ESM Fig. 4a, b). Taken together, these results indicate that the SUR1 R1420H variation does not alter PP generation.

### Immature SUR1 1420HH and 1420RH SC-islets show hyperinsulinaemia and are responsive to diazoxide

**Fig. 2:**
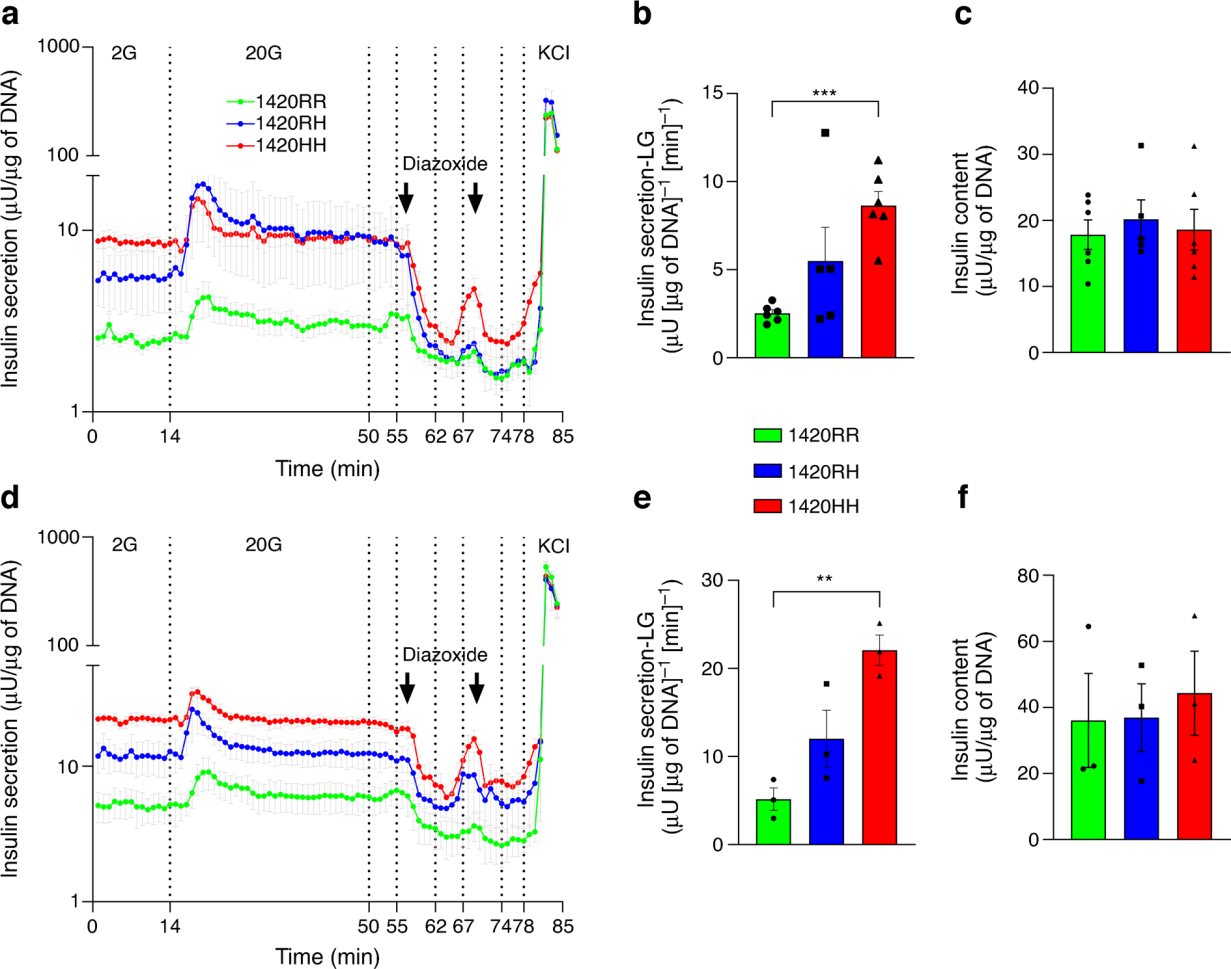
SUR1 1420HH and 1420RH immature SC-islets display insulin hypersecretion during basal glucose conditions and are diazoxide responsive (see also ESM Fig. 7). (**a**, **d**) Insulin secretion from immature SC-islets (S6D7) during d-GSIS assays (perifusion). IS1 differentiations 1–4 (**a**) (green line, 1420RR [*n*=6]; blue line, 1420RH [*n*=5]; red line, 1420HH [*n*=6]) and IS2 differentiations 5–7 (**d**) (green line, 1420RR; blue line, 1420RH; red line, 1420HH [all *n*=3]). Conditions were as follows: minutes 1–14, 2 mmol/l glucose (2G); minutes 15–50, 20 mmol/l glucose (20G); minutes 51–55, 2 mmol/l glucose; minutes 56–62, 2 mmol/l glucose + 100 µmol/l diazoxide; minutes 63–67, 2 mmol/l glucose; minutes 68–74, 20 mmol/l glucose + 100 µmol/l diazoxide; minutes 75–78, 2 mmol/l glucose; minutes 79–85, 2 mmol/l glucose + 30 mmol/l KCl. Data are presented as mean ± SEM. (**b**, **e**) Basal insulin secretion (during minutes 1–12) from IS1 immature SC-islets (**b**) and IS2 immature SC-islets (**e**). **p<0.01, ***p<0.001. Data are presented as mean ± SEM. *n*, as for (**a**) and (**d**), respectively. (**c**, **f**) Total insulin content in the IS1 immature SC-islets (**c**) and IS2 immature SC-islets (**f**) used for the perifusion assays. Data are presented as mean ± SEM. *n*, as for (**a**) and (**d**), respectively. LG, low glucose (2 mmol/l glucose)

The PP cells were further differentiated towards the pancreatic endocrine lineage; by day 20 (stage 6 day 7 [S6D7]) of differentiation, we observed a robust increase in the expression of islet-specific genes including *INS*, *GCG* and *SST* (ESM Fig. 5a, b). The differentiation efficiency was comparable between the three genotypes (Fig. 1c and ESM Figs 5, 6). These S6D7 immature SC-islets are non-glucose responsive and are comparable with fetal islets [29]. Because the one individual carrying SUR1 1420HH had HHI and individuals with the 1420RH genotype had higher birthweights suggestive of fetal hyperinsulinaemia, we used these immature SC-islets to model the effects of R1420H on insulin secretion in utero and during infancy. A d-GSIS assay confirmed that these immature SC-islets did not have a strong response to high glucose (20 mmol/l) but were responsive to KCl (30 mmol/l) (Fig. 2a, d). Immature 1420HH SC-islets had 3.4-fold (IS1, Fig. 2b, *p*<0.001) and 4.2-fold (IS2, Fig. 2e, *p*=0.001) higher insulin secretion rates in low glucose (2 mmol/l) than the 1420RR immature SC-islets despite having a similar insulin content (IS1, Fig. 2c; IS2, Fig. 2f). Immature 1420RH SC-islets also had higher insulin secretion rates than 1420RR SC-islets but this difference was not statistically significant (IS1, 2.2-fold, *p*=0.12; IS2, 2.3-fold, *p*=0.12). Similar results were seen when data were normalised to total insulin content (ESM Fig. 7). We then assessed whether immature SC-islets with the His-allele were diazoxide responsive and, interestingly, immature SC-islets (both IS1 and IS2) with all three genotypes responded to diazoxide by reducing insulin secretion in both low and high glucose conditions (Fig. 2a, d). This suggests that this model could be used for screening therapeutic agents that may prevent HHI and that infants homozygous for the SUR1 R1420H variation would benefit from diazoxide use to reduce their hyperinsulinaemia.

### SC-islet maturation, composition and hormone content

**Fig. 3:**
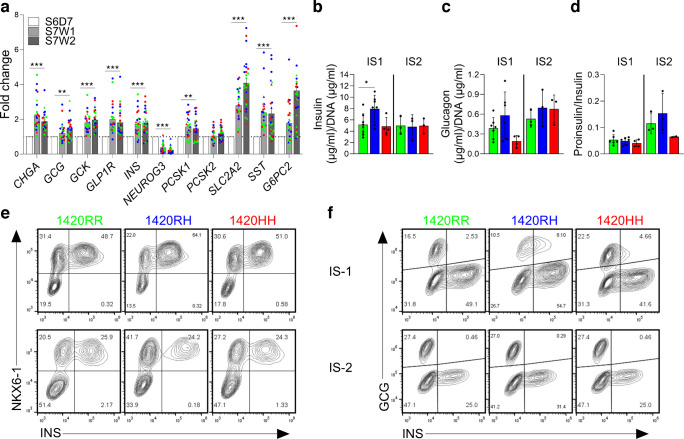
SC-islet maturation, composition and hormone content (see also ESM Figs 8 and 9). (**a**) Fold change in mRNA expression of select maturation markers at S7W1 (day 27) and S7W2 (day 34) of differentiation relative to mRNA expression levels in immature SC-islets (S6D7). IS1 cells (differentiations 2–6): green circles, 1420RR (*n*=7); blue circles, 1420RH (*n*=6); red circles, 1420HH (*n*=4). IS2 cells (differentiations 5–7): green triangles, 1420RR; blue triangles, 1420RH; red triangles, 1420HH (all *n*=3). **p<0.01 and ***p<0.001 for S6D7 vs S7W2 cells after correction for multiple testing (Šídák–Bonferroni method). Data are presented as mean ± SD. Two data points (*G6PC2* 1420RH and *G6PC2* 1420HH >8.0) are not represented in the figure for scaling purposes. (**b**) Insulin content in S7W2 mature SC-islets. IS1 cells (differentiations 1–6): green bars, 1420RR (*n*=8); blue bars, 1420RH (*n*=7); red bars, 1420HH (*n*=4). IS2 cells (differentiations 5–7): green bars, 1420RR; blue bars, 1420RH; red bars, 1420HH (all *n*=3). *p<0.05. Data are presented as mean ± SD. (**c**, **d**) Glucagon content (**c**) and proinsulin/insulin ratio (**d**) in S7W2 mature SC-islets. IS1 cells (differentiations 2–6): green bars, 1420RR (*n*=7); blue bars, 1420RH (*n*=6); red bars, 1420HH (*n*=4). IS2 cells (differentiations 5–7): green bars, 1420RR; blue bars, 1420RH; red bars, 1420HH (all *n*=3). Data are presented as mean ± SD. (**e**, **f**) Representative flow cytometry plots for INS/NKX6-1 staining (**e**) and INS/GCG staining (**f**) in mature SC-islets (S7W2, day 34) generated from IS1 and IS2 1420RR, 1420RH and 1420HH cell lines. Representative plots for INS/NKX6-1 and INS/GCG from different differentiations are shown

To model the temporal changes in insulin secretion due to the SUR1 R1420H variation after childbirth and during adulthood, we further differentiated the S6D7 immature SC-islets to a more adult-like mature stage (stage 7 week1 [S7W1] and stage 7 week 2 [S7W2]). We first compared the maturation efficiency by assessing the expression of maturation markers, hormone content and islet composition in these mature SC-islets. During S7W1 and S7W2, there was a significant upregulation in the expression of islet maturation marker genes (*GCK*, *GLP1R*, *PCSK1*, *SLC2A2* and *G6PC2*) and hormone genes (*INS*, *GCG* and *SST*) relative to S6D7 immature SC-islets, while *NEUROG3* expression (essential for endocrine commitment of PP cells) was significantly reduced (Fig. 3a). The change in gene expression in S7W1 and S7W2 mature SC-islets relative to immature SC-islets did not differ by genotype (ESM Fig. 8). The insulin and glucagon contents of S7W2 SC-islets were comparable with those of adult islet preparations [17] and had a favourable proinsulin/insulin ratio (<0.1 for IS1 and <0.2 for IS2) indicating proper insulin processing, and differed by genotype only in IS1 1420RH SC-islets wherein a significantly higher insulin content (difference in mean ± SEM=2.8 ± 1.1 µg of insulin/µg of DNA, *p*=0.03) was observed compared with IS1 normal SC-islets (Fig. 3b–d).

The proportion of functional SC-beta cells (INS^+^NKX6-1^+^ cells) was also not statistically different among the isogenic SC-islets with the three genotypes (ESM Fig. 9c, e); however, there was a notable difference when comparing the proportion of SC-beta cells between the IS1 and IS2 cell lines (median, IS1: 1420RR=47.6%; 1420RH=59.5%; 1420HH=51%; and median, IS2: 1420RR=24.2%; 1420RH=27.2%; 1420HH=23.3%) (Fig. 3e, f and ESM Fig. 9g, h). The Indigenous community from which the IS1 and IS2 cell lines were derived is known to have common variation in the *KCNQ1* gene, a well-established locus for type 2 diabetes, where more than half of the community carries *KCNQ1* risk alleles that affect beta cell mass and have a notable impact on type 2 diabetes risk [22, 30, 31]. Sanger sequencing across the *KCNQ1* region in the IS1 and IS2 parental lines revealed that IS1 is homozygous for the *KCNQ1* non-risk alleles and IS2 is heterozygous for the *KCNQ1* type 2 diabetes risk alleles. This suggests that the difference in SC-beta cell proportion may be driven by key genomic variability (e.g. variants in the *KCNQ1* locus) between IS-1 and IS-2 rather than experimental variability, underscoring the importance of comparing isogenic cell lines to avoid bias introduced by additional genomic variation between individuals. The proportion of GCG^+^ cells were also similar among the SC-islets with the three genotypes (ESM Fig. 9d, f). Taken together, these results indicate proper generation of mature SC-islets with all three genotypes.

### SUR1 1420HH and 1420RH SC-islets have lower or no insulin secretory response to glucose and tolbutamide

**Fig. 4:**
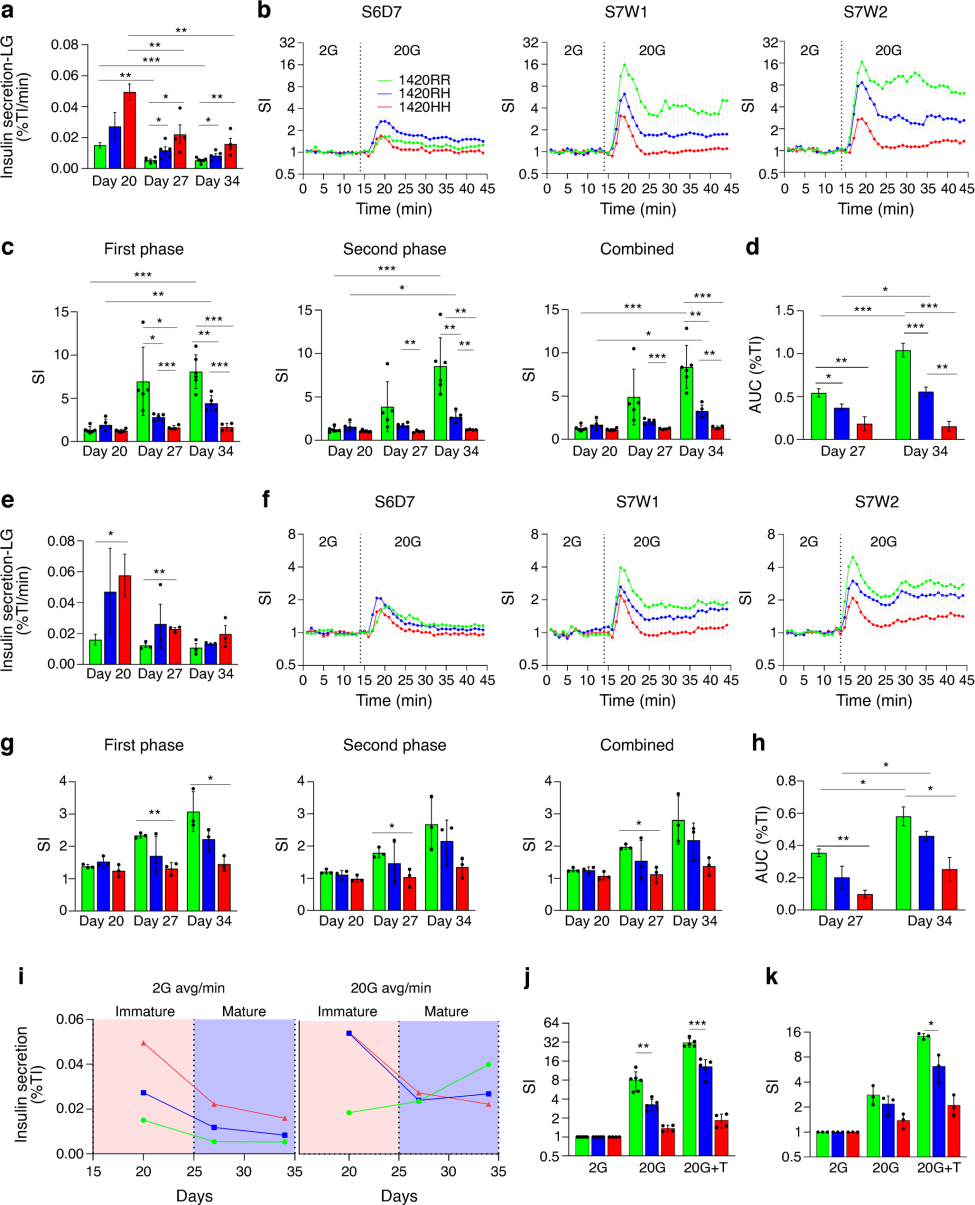
SUR1 1420HH and 1420RH mature SC-islets have a lower insulin secretory response to glucose and tolbutamide (see also ESM Fig. 10). Insulin secretion during d-GSIS assays (perifusion) from mature SC-islets (S7W1 and S7W2). Conditions were as follows: minutes 1–14, 2 mmol/l glucose (2G); minutes 15–44, 20 mmol/l glucose (20G); and minutes 45–50, 20G + 100 µmol/l tolbutamide. IS1-S7W1 (differentiations 1–4): 1420RR, *n*=5; 1420RH, *n*=5; 1420HH, *n*=4. IS1-S7W2 (differentiations 1–4): 1420RR, *n*=6; 1420RH, *n*=5; 1420HH, *n*=4. IS2-S7W1 and -S7W2 (differentiations 5–7): 1420RR, *n*=3; 1420RH, *n*=3; 1420HH, *n*=3. For (**a**–**k**): green, 1420RR; blue, 1420RH; red, 1420HH. *p<0.05, **p<0.01, ***p<0.001. (**a**, **e**) Insulin secretion/min during the first 12 min (2G) from IS1 SC islets (**a**) and IS2 SC-islets (**e**) at S7W1 (day 27) and S7W2 (day 34). Insulin secretion from S6D7 immature SC-islets (day 20) is also shown. Data are presented as mean ± SEM. (**b**, **f**) Biphasic insulin secretory response to 20G, shown as SI (fold increase in insulin secretion compared with secretion when stimulated with 2G [first 12 min]), during the first 44 min (minutes 1–14 with 2G and minutes 15–44 with 20G) using S7W1 (day 27) and S7W2 (day 34) IS1 SC-islets (**b**) and IS2 SC-islets (**f**). Data are presented as mean ± SEM. Insulin secretory response from S6D7 (day 20) immature SC-islets is also shown for comparison. (**c**, **g**) SI during first-phase (minutes 15–24), second-phase (minutes 25–44) and combined stimulation with 20G at S6D7, S7W1 and S7W2 from IS1 SC-islets (**c**) and IS2 SC-islets (**g**). Data are presented as mean ± SD. (**d**, **h**) Insulin secretion as AUC during 20G stimulation using S7W1 and S7W2 IS1 SC-islets (**d**) and IS2 SC-islets (**h**). Data are presented as mean ± SEM. (**i**) insulin secretion from immature and mature IS1 SC-islets with the three genotypes when stimulated with either 2G or 20G. (**j**, **k**) Insulin secretory response (SI) to tolbutamide (T) from S7W2 IS1 SC-islets (**j**) and IS2 SC-islets (**k**). Data are presented as mean ± SD. Avg, average; %TI, percent of total insulin

Next, we performed d-GSIS assays to assess insulin secretory responses from these S7W1 (day 27) and S7W2 (day 34) mature SC-islets. A significant reduction in basal insulin secretion (2 mmol/l stimulation) was observed in IS1 mature SC-islets (S7W2) compared with immature SC-islets (2.9-fold reduction in 1420RR [*p*<0.001], 3.3-fold in 1420RH [*p*=0.07] and 3.1-fold in 1420HH [*p*=0.001] mature SC-islets); however, the 1420RH (1.5-fold, *p*=0.05) and 1420HH (3.0-fold, *p*=0.008) mature SC-islets had a greater basal insulin secretion rate than normal 1420RR SC-islets (Fig. 4a and ESM Fig. 10a, b). Increasing the glucose concentration from 2 mmol/l to 20 mmol/l resulted in a biphasic insulin secretory response from both S7W1 and S7W2 1420RR and 1420RH SC-islets but only a weak first-phase response from 1420HH SC-islets. 1420RR SC-islets had stronger response to glucose than either 1420RH or 1420HH SC-islets (Fig. 4b and ESM Fig. 10c, d). No significant increase in either the first- or the second-phase insulin secretory response was seen in 1420HH SC-islets after maturation (day 27 and day 34, Fig. 4c), while both the normal 1420RR SC-islets (first-phase SI=8.1, second-phase SI=8.5) and 1420RH SC-islets (first-phase SI=4.4, second-phase SI=2.7) had a significantly higher SI after maturation (S7W2, day 34) compared with immature SC-islets (Fig. 4c; 1420RR, *p*<0.001 for both first and second phases; 1420RH, *p*=0.001 for first phase and *p*=0.01 for second phase). The AUC for insulin secretion from mature SC-islets during the high glucose phase increased from S7W1 to S7W2 for the 1420RR SC-islets (1.9-fold increase, *p*<0.001) and 1420RH SC-islets (1.5-fold increase, *p*=0.03) but there was no increase for 1420HH SC-islets (Fig. 4d). The corresponding AUC was significantly greater for the 1420RR SC-islets compared with 1420RH SC-islets at both S7W1 (0.54 vs 0.37 [insulin secretion AUC as a % of total insulin], *p*=0.03) and S7W2 (1.04 vs 0.56, *p*<0.001) (Fig. 4d,) suggesting an impaired insulin secretory response to glucose from 1420RH SC-islets as well.

Consistent with a lower proportion of functional beta cells, the IS2 SC-islets also had lower insulin secretory response to glucose. However, directionally comparable results were seen from these SC-islets as well when comparing the effect of the SUR1 R1420H variation on insulin secretory response to glucose (Fig. 4e–h and ESM Fig. 10e–h).

Our d-GSIS results suggest that the 1420RH and 1420HH SC-islets have higher insulin secretion in response to both low glucose and high glucose when they are immature, and upon maturation there is a tighter regulation of insulin secretion in low glucose condition (Fig. 4i). However, in contrast to 1420RR SC-islets, the 1420RH and 1420HH SC-islets secrete a lower per cent of their insulin content and either have lower or no insulin secretory response, respectively, to glucose challenge after maturation (Fig. 4i). These results provide experimental evidence that an insulin secretory defect is the primary cause for the higher type 2 diabetes risk in individuals with the SUR1 1420RH variation.

We then tested whether our model could be used for testing therapeutic agents for improvement of insulin secretory response from islets with the K_ATP_ channel LoF variations. For this, we assessed the effect of the K_ATP_ channel blocker tolbutamide on insulin secretion from mature SC-islets. As expected, the 1420RR SC-islets had increased insulin secretion (SI=31.5), while the 1420RH (SI=13.2) and 1420HH SC-islets (SI=1.9) had a significantly lower or no response to tolbutamide (Fig. 4j, k and ESM Fig. 10).

### Heterozygous 1420RH SC-islets have blunted response to increasing glucose concentration

**Fig. 5:**
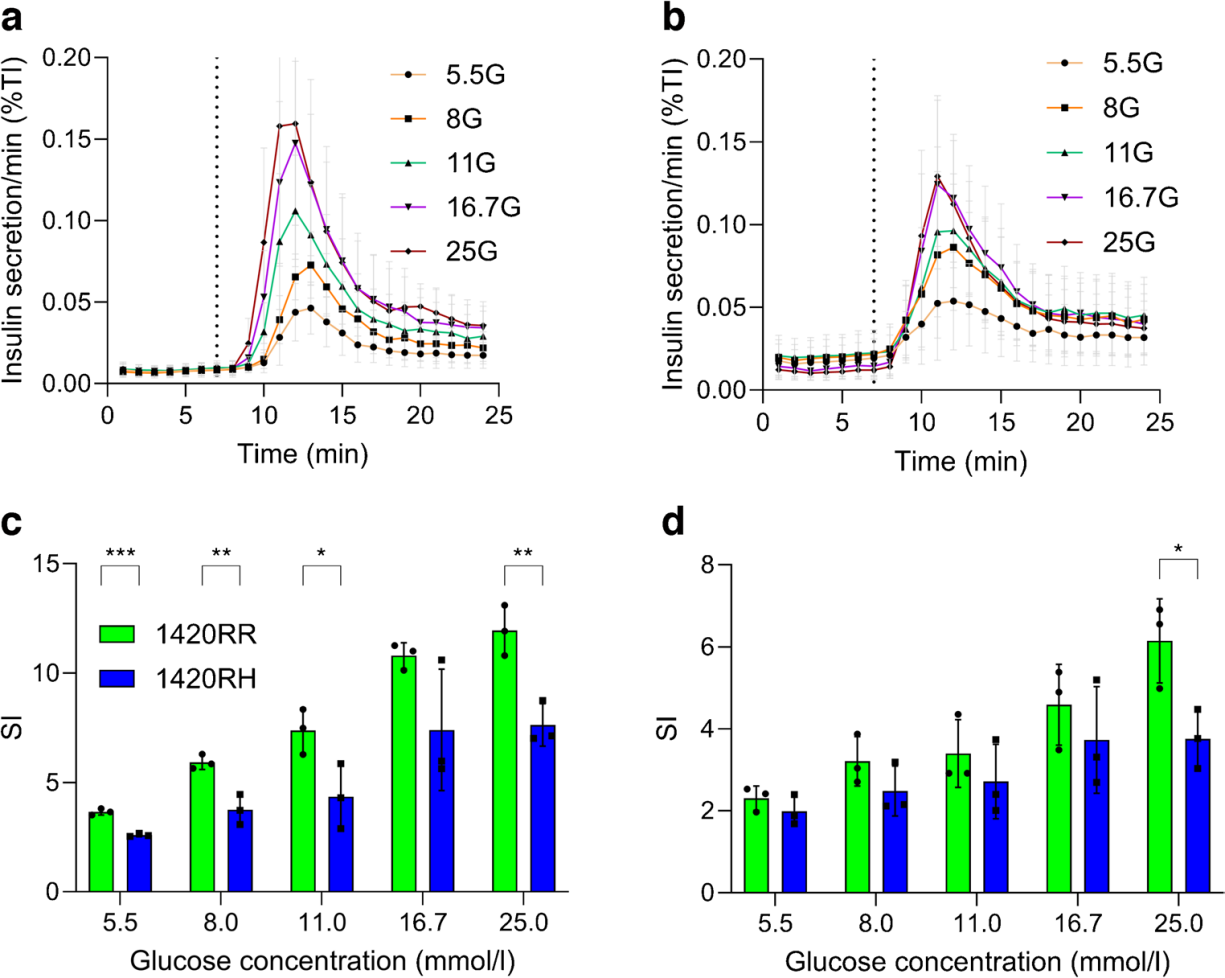
Mature SUR1 1420RH SC-islets have a blunted response to increasing glucose concentrations (see also ESM Fig. 11). S7W2 IS1 SC-islets (days 35–40, differentiations 3–5) were used for testing the effect of different glucose concentrations on the insulin secretory response by d-GSIS. The flow-through was collected every minute and assayed for insulin by ELISA. (**a**, **b**) Insulin secretory responses to different glucose concentrations from normal 1420RR (**a**, *n*=3) and heterozygous 1420RH (**b**, *n*=3) SC-islets. SC-islets were perifused with 2 mmol/l glucose (2G, minutes 1–7) followed by 5, 8, 11, 16.7 or 25 mmol/l glucose (minutes 8–24). Data are presented as mean ± SEM. (**c**, **d**) SI during first-phase (**c**, minutes 9–16) and second-phase (**d**, minutes 17–24) insulin secretion from normal 1420RR (green, *n*=3) and heterozygous 1420RH (blue, *n*=3) SC-islets stimulated with different glucose concentrations. Data are presented as mean ± SD. *p<0.05, **p<0.01, ***p<0.001

Immature heterozygous 1420RH SC-islets display mild hyperinsulinaemia but after maturation have lower insulin secretory response to maximal glucose. Therefore, we examined the response of these SC-islets to a range of glucose concentrations. When the mature 1420RH SC-islets were challenged with a range of glucose concentrations (5.5–25 mmol/l), we observed a blunted insulin secretory response to increasing glucose concentrations compared with the 1420RR SC-islets (Fig. 5a, b and ESM Fig. 11). The first-phase SI was significantly lower for the 1420RH SC-islets at all glucose concentrations, with larger differences seen at higher concentrations compared with 1420RR SC-islets (Fig. 5c and ESM Fig. 11m, n) while the second-phase response was significantly different only at 25 mmol/l glucose concentration (Fig. 5d and ESM Fig. 11o).

### Heterozygous 1420RH SC-islets respond to GCK activator dorzagliatin

**Fig. 6:**
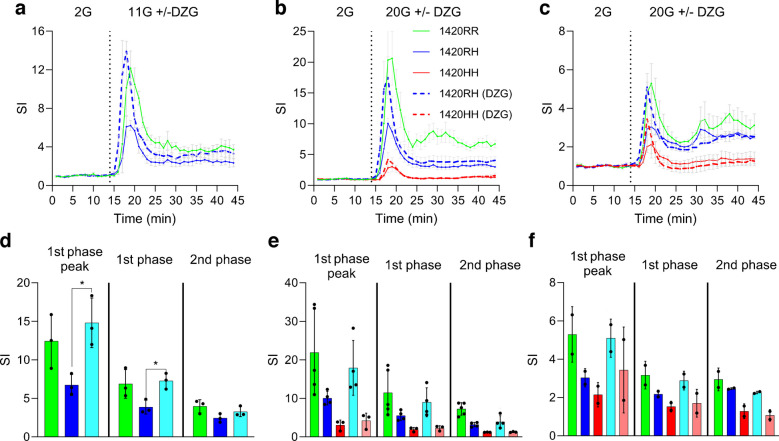
Mature 1420RH SC-islets respond to dorzagliatin. For testing the effect of dorzagliatin, S7W2 IS1 SC-islets (**a**, **b**, **d**, **e**) and IS2 SC-islets (**c**, **f**) were used. SC-islets were perifused with KRB containing 2 mmol/l glucose (2G, minutes 1–14) followed by 11 mmol/l glucose (11G) or 20 mmol/l glucose (20G, minutes 15–44) with or without 25 µmol/l dorzagliatin (DZG). The flowthrough was assessed for insulin secretion every minute. (**a**) Insulin secretory response (SI) from S7W2 IS1 heterozygous 1420RH SC-islets (differentiations 4–6) when treated with 11G glucose in the presence (dashed blue line) or absence (solid blue line) of DZG. The response from normal SC-islets (1420RR, green line) in the absence of DZG is shown as a reference. 1420RR, *n*=3; 1420RH, *n*=3. Data are presented as mean ± SEM. (**b**, **c**) Insulin secretory response from S7W2 IS1 (**b**) and IS2 (**c**) heterozygous 1420RH and homozygous 1420HH SC-islets when treated with 20G glucose in the presence (dashed blue line, 1420RH; dashed red line, 1420HH) or absence (solid blue line, 1420RH; solid red line, 1420HH) of DZG. The response from normal SC-islets (1420RR, green line) in the absence of DZG is shown as a reference. IS1 (differentiations 3–6): 1420RR, *n*=5; 1420RH, *n*=4; 1420HH, *n*=3. IS2 (differentiation 6 and 7): 1420RR, *n*=2; 1420RH, *n*=2; 1420HH, *n*=2. Data are presented as mean ± SEM. (**d**) First-phase peak, first-phase (minutes 15–22) and second-phase (minutes 23–44) SI during d-GSIS using S7W2 IS1 heterozygous 1420RH SC-islets (*n*=3) challenged with 11G in the presence (light blue bar) or absence (blue bar) of DZG. The SI of normal SC-islets (1420RR, *n*=3, green bar) in the absence of DZG is shown as a reference. Data are presented as mean ± SD. (**e**, **f**) First-phase peak, first-phase (minutes 15–22) and second-phase (minutes 23–44) SI during d-GSIS using S7W2 IS1 (**e**) and IS2 (**f**) heterozygous 1420RH and homozygous 1420HH SC-islets challenged with 20G in the presence (light blue bars, 1420RH; light red bars, 1420HH) or absence (blue bars, 1420RH; red bars, 1420HH) of DZG. The SI of normal SC-islets (1420RR, green bars) in the absence of DZG is shown as a reference. IS1 (differentiations 3–6): 1420RR, *n*=5; 1420RH, *n*=4; 1420HH, *n*=3. IS2 (differentiation 6 and 7): 1420RR, *n*=2; 1420RH, *n*=2; 1420HH, *n*=2. Data are presented as mean ± SD. *p<0.05

The lower insulin secretory response to increasing glucose concentrations could be due to reduced glycolytic flux resulting in lower ATP production and lower insulin secretion. GCK acts as the primary glucose sensor and its activity is a crucial determinant of ATP production in pancreatic beta cells [32]. To examine whether activation of GCK in the 1420RH SC-islets could improve insulin secretory response to glucose, the SC-islets were treated with the GCK activator dorzagliatin (25 μmol/l) during d-GSIS. Stimulation with high glucose in the presence of dorzagliatin resulted in a slight leftward shift of the first-phase insulin secretory curve, indicative of GCK activation (Fig. 6a–c). Dorzagliatin, in the presence of 11 mmol/l glucose (Fig. 6a, d, IS1 cells) or 20 mmol/l glucose (Fig. 6b, e, IS1 cells) resulted in improved first-phase insulin secretory response from 1420RH SC-islets (first-phase SI=7.3-fold and 3.9-fold [*p*=0.01] with and without dorzagliatin + 11 mmol/l glucose, respectively [Fig. 6d]; first-phase SI=9.0-fold and 5.5-fold [*p*=0.13] with and without dorzagliatin + 20 mmol/l glucose, respectively [Fig. 6e]). In contrast, dorzagliatin had a minimal effect on non-glucose-responsive 1420HH SC-islets (first-phase SI=2.3-fold and 1.9-fold with and without dorzagliatin + 20 mmol/l glucose, respectively [Fig. 6e]). Similar results were seen with mature SC-islets generated from IS2 cell lines (that have a lower proportion of SC-beta cells) as well when challenged with 20 mmol/l glucose in the presence of dorzagliatin (Fig. 6c, f).

### Transcriptome comparison of individual SC-islet cell types by SUR1 R1420H

**Fig. 7:**
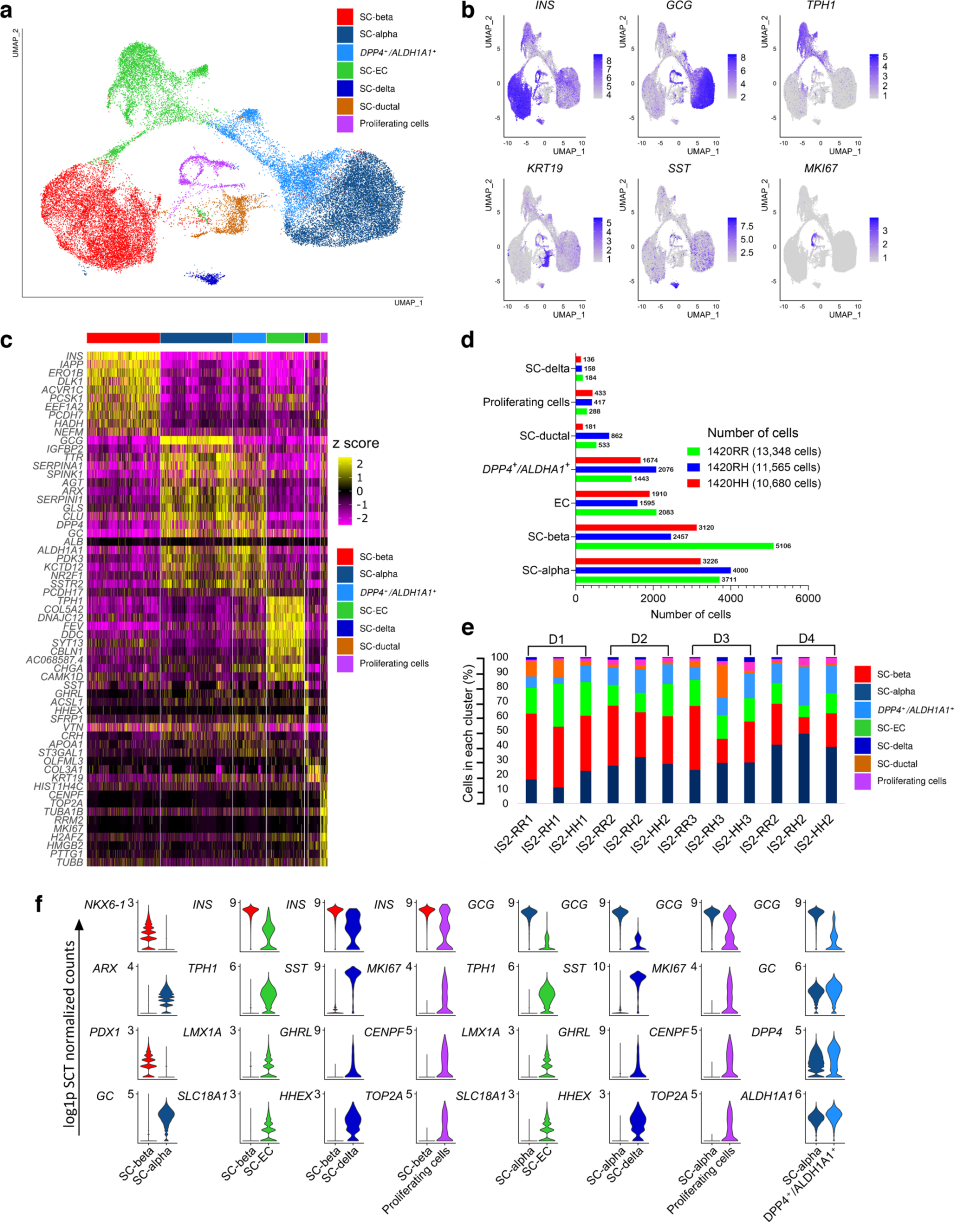
Single-cell transcriptome analyses of S7W4 (day 48) IS2 SC-islets (see also ESM Table 5). (**a**) UMAP of 35,593 cells clustered and colour coded to indicate seven distinct cell types. (**b**) Feature plots showing expression (log1P SCT normalised counts) of representative marker genes for each cell type (*INS*, *GCG*, *TPH1*, *KRT19*, *SST*, *MKI67*). (**c**) Scaled expression heatmap showing the top differentially expressed genes (DEGs) for each cluster compared with all other clusters. (**d**) Bar graph showing the number of cells in each cluster by genotype. (**e**) Bar graph showing the percentage of cells in each cluster by genotype/clone and by different differentiations (IS2 differentiations 1–4). (**f**) Violin plots showing expression (log1P SCT normalised counts) of core markers of other islet cell types in the designated SC-alpha and SC-beta cluster. EC, enterochromaffin

We next looked at the transcriptomic changes that may explain the lower/no insulin secretory response to glucose in mature 1420RH and 1420HH SC-islets. Due to the known heterogeneity of islets (derived directly from humans or generated as SC-islets), we employed single-cell RNA-seq (scRNA-seq) to compare transcriptomes of pure population of SC-beta and SC-alpha cells from S7W4 (day 48) mature SC-islets with all three genotypes (ESM Fig. 12). We acquired scRNA-seq data from 35,593 cells generated from four independent differentiations using nine IS2 cell lines. We used Seurat for data processing and clustering and performed an unbiased differential gene expression analysis. The top 100 differentially expressed genes in each cluster was used for cell type assignment and these were consistent with the core identity genes for SC-beta, SC-alpha, iPSC-derived delta (SC-delta) and enterochromaffin cell types as reported by Schmidt et al [33] (see ESM Table 5). The identified SC-islet cell types included SC-alpha cells (30.7%), SC-beta cells (30.01%), duct-like cells (4.4%), SC-delta cells (1.3%) and proliferating cells (3.2%) (Fig. 7a–c). There was also a population of *DPP4*^+^/*ALDH1A1*^+^ cells (14.6%) that expressed several alpha cell markers but did not express *GCG*; however, this population did not differ by genotype and was classified as *DPP4*^+^/*ALDH1A1*^+^ cells (Fig. 7d, e). Consistent with previous single-cell sequencing reports of SC-islets, we also observed a population of cells that were positive for *INS* but expressed the enterochromaffin markers *TPH1* and *FEV* (Fig. 7a–c). These cells were classified as enterochromaffin cells (15.7%). A higher number and proportion of SC-beta cells were recovered from 1420RR SC-islets after standard quality control (38.2% in 1420RR vs 21.2% in 1420RH vs 29.2% in 1420HH) but this difference was not statistically significant and was not consistent among the four separate differentiations (Fig. 7d, e). The proportions of the other cell types recovered from the four differentiations were also not statistically different between the normal SC-islets and K_ATP_ channel variant SC-islets (Fig. 7e and ESM Fig. 13a); however, we did observe a greater proportion of *MKI67*^+^ proliferating cells in SC-islets with the K_ATP_ channel variation (2.16% vs 3.6% vs 4.05% in 1420RR, 1420RH and 1420HH SC-islets, respectively). Islets generated from iPSCs may also contain polyhormonal SC-beta and SC-alpha cells (ESM Fig. 13b) that co-express *INS* and *GCG* [33]. We identified a small percentage (3.5%) of *INS*^*+*^*/GCG*^*+*^ cells and the distribution of these polyhormonal cells did not differ by genotype. In total, our clustering analysis identified 10,683 SC-beta cells and 10,937 SC-alpha cells that expressed core beta cell and alpha cell identity markers and had low expression of core markers of other SC-islet cell types (Fig. 7f). Before further analysis, *INS*^+^/*GCG*^+^ polyhormonal cells were removed from both the SC-beta and the SC-alpha cluster resulting in 10,621 SC-beta cells and 10,232 SC-alpha cells.

### Differentially expressed genes in SC-beta cells and SC-alpha cells with R1420H K_ATP_ channel variation

**Fig. 8:**
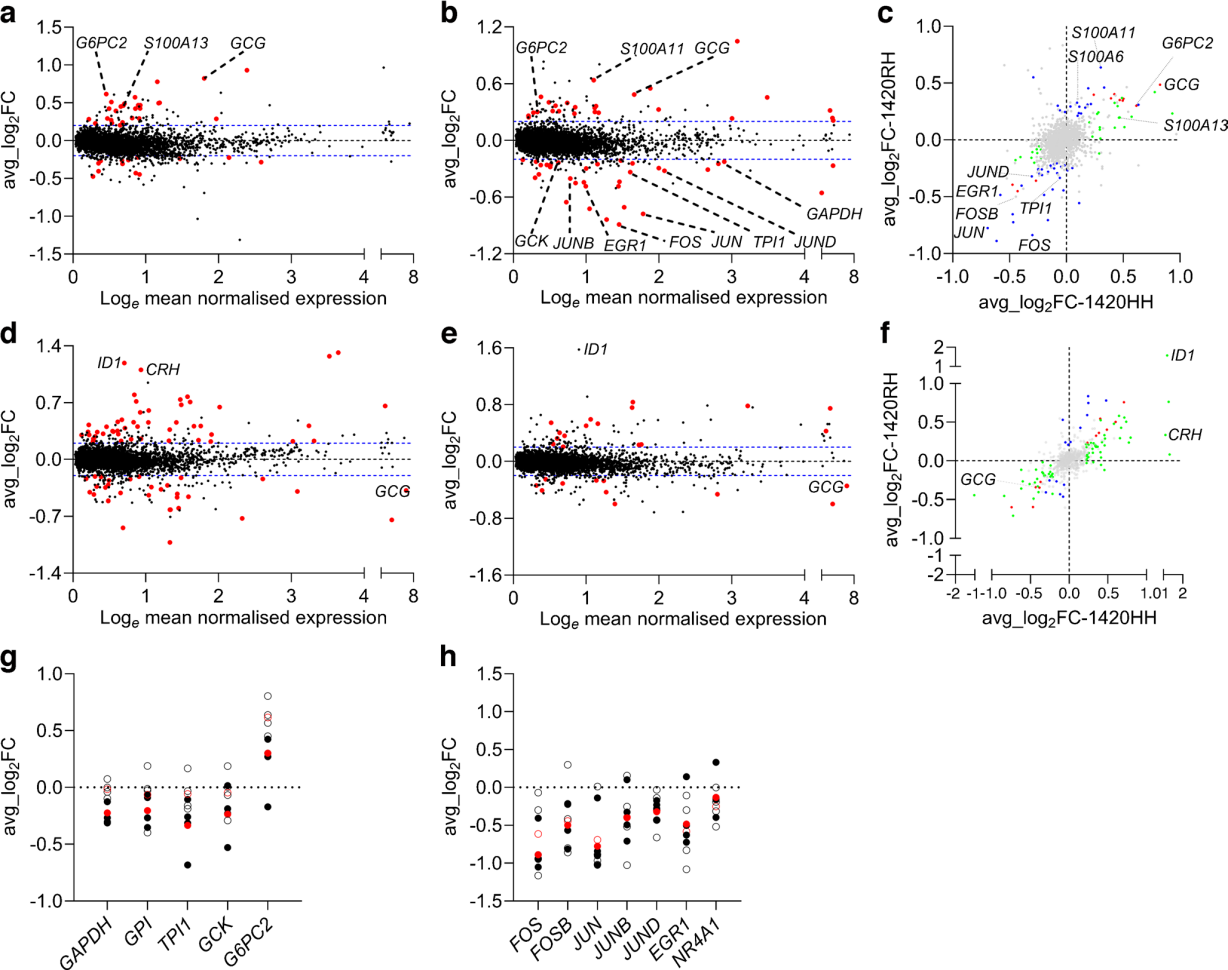
Dysregulated genes in SC-beta and SC-alpha cells with SUR1 1420RH and 1420HH variation (see also ESM Tables 6–10). Data from single-cell sequencing of SC-islets from four independent differentiations using nine isogenic IS2 cell lines (three cell lines for each genotype). (**a**, **b**, **d**, **e**) MA plots showing upregulated and downregulated genes in homozygous 1420HH SC-beta cells (**a**) and SC-alpha cells (**d**) and in heterozygous 1420RH SC-beta cells (**b**) and SC-alpha cells (**e**) compared with normal 1420RR SC-beta and SC-alpha cells, respectively. Dysregulated genes are highlighted as larger red circles. Three data points are not represented in the figure for scaling purposes: *MEG3* in (**b**); and *NPW* and *SST* in (**d**). (**c**, **f**) Modified four-way plots comparing the log_2_FC of all significant genes (sample adjusted *p*<0.05) in homozygous 1420HH vs heterozygous 1420RH SC-beta cells (**c**) and homozygous 1420HH vs heterozygous 1420RH SC-alpha cells (**f**). Dysregulated genes that are directionally consistent in both 1420HH and 1420RH are in the upper right and lower left quadrants. Red circles, genes identified as dysregulated in both 1420HH and 1420RH cells; green circles, genes identified as dysregulated in only 1420HH cells; blue circles, genes identified as dysregulated in only 1420RH cells. (**g**, **h**) Log_2_FC of glycolytic genes and *G6PC2* (**g**) and immediate early response genes (**h**) in SC-beta cells from individual differentiations (black) and combined analysis (red) from the homozygous 1420HH vs normal 1420RR SC-beta cell differential gene expression analysis (open circles) and the heterozygous 1420RH vs normal 1420RR SC-beta cell differential gene expression analysis (closed circles). Avg, average

Next, differential gene expression comparisons were performed for both SC-beta and SC-alpha cells separately as follows: 1420HH vs 1420RR cells; and 1420RH vs 1420RR cells. To account for differences in gene expression due to variation in differentiation efficiency occurring in samples with the same R1420H genotype, ‘sample’ was used as a latent variable. We identified 36 and 75 upregulated (*p*<0.05, log_2_ fold change [log_2_FC] >0.2) and 30 and 42 downregulated genes (*p*<0.05, log_2_FC <−0.2) in 1420HH SC-beta and SC-alpha cells, respectively (ESM Table 6). In 1420RH SC-beta and SC-alpha cells, 61 and 40 upregulated and 75 and 29 downregulated genes were identified, respectively (ESM Table 6). Alternatively, we took advantage of the fact that the cells were sourced from different differentiations and were generated using different clonal cell lines for each genotype. Differential gene expression analysis was performed separately for each of the four differentiations and genes that were significant in at least three of the four differentiations and the combined analysis (without adjustment for ‘sample’, log_2_FC >0.2 or log_2_FC < −0.2) were identified (ESM Tables 7, 8). Only genes that were common in both analyses were considered dysregulated (shown as enlarged red circles in Fig. 8a, b, d, e; see also ESM Tables 7, 8).

In total, we identified 40 and 81 genes in the 1420HH SC-beta and SC-alpha cells, respectively, that were dysregulated, while 59 and 27 dysregulated genes were identified in 1420RH SC-beta and SC-alpha cells, respectively. The majority of the dysregulated genes were directionally consistent (upregulated in both 1420HH and 1420RH cells or downregulated in both 1420HH and 1420RH cells) and only few discrepant dysregulated genes were observed in both SC-beta and SC-alpha cells (Fig. 8c, f and ESM Tables 9, 10). Interestingly, *GCG* was dysregulated in all four comparisons but was discrepant between SC-beta and SC-alpha cells; *GCG* was directionally consistent and downregulated in both 1420HH and 1420RH SC-alpha cells but upregulated in both 1420HH and 1420RH SC-beta cells (Fig. 8c, f).

Consistent with our previous results, genes involved in glucose metabolism were dysregulated (Fig. 8g). Key glycolytic genes, *GCK*, *GAPDH*, *GPI* and *TPI1* were downregulated in 1420RH SC-beta cells while *G6PC2*, a pancreatic beta cell-specific isoform of glucose 6-phosphatase catalytic subunit, which opposes the action of GCK and negatively regulates insulin secretion, was upregulated in both 1420RH and 1420HH SC-beta cells (Fig. 8g). Although bulk RNA data from islets are not always consistent with single-cell sequencing results due their inherent heterogenous nature, we also observed directionally consistent and significant changes in gene expression of *G6PC2*, *GCK* and *GAPDH* in real-time PCR using RNA from independent differentiations (ESM Fig. 14). Among the other consistently dysregulated genes in SC-beta cells were the members of the S100 family, *S100A13*, *S100A6* and *S100A11*, which are involved in Ca^+^ signalling/binding and the immediate early response genes, *FOS*, *FOSB*, *JUN*, *JUNB*, *JUND*, *EGR1* and *NR4A1* (Fig. 8a–c, h). Among the SC-alpha cell dysregulated genes, the top gene was *ID1*, which was upregulated in K_ATP_ channel variant alpha cells. *CRH*, a human alpha cell gene [34] was also one of the top upregulated genes in 1420HH SC-alpha cells (Fig. 8d) and was consistently upregulated in all four differentiations (ESM Table 8). *CRH* was also upregulated in the 1420RH SC-alpha cells, but the effect was smaller and was only significant in two out of four differentiations. Real-time PCR for both *S100A13* and *CRH* from independent differentiations were directionally consistent but not statistically significant (ESM Fig. 14).

## Discussion

We have previously reported that Indigenous individuals living in Arizona who carry the SUR1 R1420H variation either develop HHI or are at increased risk for type 2 diabetes [11]. In this study, we modelled the impact of the SUR1 R1420H variation on insulin secretion using immature SC-islets, which represent fetal islets, and mature (adult) SC-islets generated from ethnic-specific isogenic iPSCs. We created an in vitro model to understand the disparate mechanisms whereby this K_ATP_ channel variation causes hyperinsulinaemia in utero and in infancy yet increases risk of type 2 diabetes, a disease characterised by impaired insulin secretion, later in life. Our protocol for generating SC-islets mimics in utero islet development, allowing us to study the effect of the variation on antenatal islet development and eliminating the need for using fetal islets. Creating isogenic iPSCs from a community whose members are affected by the SUR1 R1420H has two advantages: (1) it overcomes the unavailability of post-mortem pancreatic islets from individuals affected by this variation; and (2) functional characterisation of the variation and assessment of specific drug therapeutics can be performed in the appropriate genomic and epigenomic background.

A previous study has modelled an established LoF K_ATP_ channel variation (V187D) using iPSC-derived SC-islets and identified important aspects of its pathophysiology [13]. For example, hyperinsulinaemia in basal condition (low glucose stimulation) from mature SC-islets homozygous for the K_ATP_ channel LoF variant was successfully modelled; however, this prior study did not model the effect of the variation on insulin secretion under high glucose stimulation or in the heterozygous state. Because of a lack of protocols for generating SC-islets with stimulation–secretion coupling at the time of the previous study [13], SC-islets generated using previous protocols were limited in scope for studying them under high glucose stimulation. As noted by the authors of that report, this was not a critical shortcoming as HHI due to K_ATP_ channel LoF variation is primarily a neonatal condition and neonates lack this coupling as well [13]. However, the SUR1 R1420H variation identified in Indigenous Americans is not an established HHI variant. Only one individual with HHI was identified who was homozygous for the variation whereas 3% of the community members were heterozygous and had a twofold increased risk of type 2 diabetes [11]. Based on the known biology of K_ATP_ channels in pancreatic beta cells, the SUR1 R1420H variant is suggestive of a causal variant for HHI in the homozygous state, although this has not been modelled experimentally. Furthermore, in a small number of individuals with normal glucose tolerance (*n*=8) heterozygous for the SUR1 R1420H variation and who had insulin response data measured by an IVGTT, no statistical evidence was detected for lower insulin secretory function [11]. Therefore, we have built upon the previous report and used iPSC-based modelling to study the effect of the SUR1 R1420H variation on insulin secretion under both low and high glucose stimulation in the homozygous and, more importantly, in the heterozygous state, which is the predominant condition found in this Indigenous American population.

Our study used a modified protocol that generated mature SC-islets with appropriate biphasic responses to high glucose and therefore we were able to show higher insulin secretion in basal condition from homozygous 1420HH SC-islets both when they were immature (similar to fetal islets) and after maturation (similar to adult islets). We further show that after maturation the homozygous 1420HH SC-islets do not achieve GSIS. We also show that the heterozygous 1420RH immature SC-islets have modest hyperinsulinaemia and achieve a biphasic response to glucose after maturation but that this response is significantly lower than in normal 1420RR SC-islets. These results suggest that alterations in insulin secretion may be the primary mechanism for the increased risk of type 2 diabetes during adulthood in heterozygous carriers of the SUR1 R1420H variation.

The previous study also identified a higher proportion of SC-beta cells and higher proliferation rate in SC-islets homozygous for the V187D LoF K_ATP_ channel variation [13]. Other morphometric studies have reported conflicting evidence regarding increased beta cell mass [35–37] and in our study we did not observe a higher proportion of SC-beta cells in SUR1 R1420H variant SC-islets. However, consistent with the previous report, in our single-cell data, we observed a higher proportion of *MKI67*^+^ proliferative cells in SC-islets with the K_ATP_ channel variation. One potential explanation for this difference is that different K_ATP_ channel variants that have unique effects on K_ATP_ channel biology were used for modelling. The SUR1 V187D variant used for modelling in the previous study [13] causes a severe form of HHI in both the homozygous state and the heterozygous state, is non-responsive to diazoxide, and both homozygous and heterozygous individuals required subtotal pancreatectomy [12]. In contrast, the SUR1 R1420H variation modelled in the current study was identified in only one individual with HHI who developed diabetes at the age of 4 years and to our knowledge heterozygous carriers do not develop HHI [11]. As shown experimentally in this study, the SUR1 R1420H variation is also responsive to diazoxide.

This differences in effect may stem from the position of the respective variation in the SUR1 protein. The previously modelled V187D variant is in the transmembrane domain (TMD0) of SUR1, variations in which often lead to SUR1 trafficking defects due to misfolded protein [38, 39], and in vitro studies of the V187D variant have shown complete loss of functional K_ATP_ channel expression in the plasma membrane [12]. In contrast, the R1420H variant modelled in the current study is in the second nucleotide-binding domain (NBD2) of SUR1, where other amino acid substitutions have been identified, including the R1420C variant which has been previously shown to result in reduced affinity for MgADP and MgATP, impairment in the cooperative ATP binding between NBD2 and nucleotide-binding domain 1 (NBD1) and lower, but not complete absence of, expression of the mutant channel, all of which leads to lower K_ATP_ channel activity in beta cells [40, 41]. We have also previously studied the in vitro effects of the amino acid change resulting from SUR1 R1420H on K_ATP_ channel activity by measuring ^86^Rb^+^ efflux across the plasma membrane of COSm6 cells co-transfected with Kir6.2 and either SUR1 1420RR or SUR1 1420RH and showed lower K_ATP_ channel activity in response to metabolic inhibition [11]. Although not directly assessed in the current study, it seems likely that the R1420H, similar to R1420C, may also impair K_ATP_ channel activity by affecting MgADP and MgATP binding to NBD2. Indeed, in silico modelling with AlphaFold 3 [42] found no difference in the predicted fold of the monomer SUR1 protein with the R1420H variation while in silico molecular docking with MgADP [43] and MgATP [43] using SwissDock [44] in a predicted SUR1 R1420H model generated using SWISS-MODEL [45] suggested lower binding affinity for the variant 1420H SUR1 (see ESM Methods: Secondary structure prediction using Alphafold 3 and SWISS-MODEL for more details).

One of the basic questions that has arisen from clinical phenotyping of individuals with the SUR1 R1420H variation is the underlying mechanism for the switch in insulin secretion from hyperinsulinaemia in utero and infancy to type 2 diabetes later in life. Our model provides insights into the change in insulin secretion from SC-islets with the SUR1 R1420H variation as they mature (become more adult-like). Like normal 1420RR SC-islets, homozygous 1420HH and heterozygous 1420RH SC-islets have a tighter regulation of their basal insulin secretion after maturation. But the 1420HH SC-islets have a near total absence of insulin secretory response to glucose. This might be due to the total loss of K_ATP_ channel function. The heterozygous 1420RH SC-islets retain some K_ATP_ channel function and have a biphasic insulin secretory response to glucose after maturation, although the response is significantly lower than that of normal SC-islets and the difference is larger at higher glucose concentrations. We hypothesised that this may be due to lower glycolytic flux with increasing glucose concentration in the 1420RH SC-islets. In support of this hypothesis, our single-cell data revealed upregulation of *G6PC2* and downregulation of glycolytic genes, including *GCK*, in 1420RH SC-beta cells. A study has previously reported altered expression of glycolytic genes in K_ATP_ channel LoF human beta cells [35]. Consistent with our finding, they also reported downregulation of *GCK* in K_ATP_ channel LoF human beta cells; however, in contrast to our findings other glycolytic genes including *GAPDH* and *TPI1* were upregulated [35]. In support of the observation from our single-cell sequencing data, we saw a 4.4-fold and 3.9-fold increase in *G6PC2* expression on SC-islet maturation in 1420HH and 1420RH SC-islets, respectively, compared with a 2.8-fold increase in normal SC-islets (ESM Fig. 8) in our real-time PCR data using bulk RNA from independent differentiations.

GCK functions as the glucose sensor in beta cells and phosphorylates glucose during the first step of glycolysis [32]. It has been suggested that glucose phosphorylation rather than glucose transport into beta cells is the major control point for GSIS [46]. Moreover, it has been shown that overexpression of GCK increases GSIS [47–49] while downregulation decreases GSIS [50]. In contrast, *G6PC2* encodes a beta cell-specific glucose 6-phosphatase catalytic subunit and opposes the action of GCK by dephosphorylating glucose 6-phosphate back to glucose, creating a futile cycle and reducing glycolytic flux [51]. Studies in mice have shown that *G6pc2* can negatively regulate basal insulin secretion and that depletion of *G6pc2* leads to ~60% increase in glycolytic and TCA cycle flux in both low (5 mmol/l) and high glucose (11 mmol/l) conditions, confirming its role in GSIS [51, 52]. During SC-islet maturation, there is a significant increase in the expression of *G6PC2* concordant with a tighter regulation of basal insulin secretion (Figs 3a, 4a). Based on the known role of glucose 6-phosphatase catalytic subunit 2 as a negative regulator of basal insulin secretion, we propose a model where hyperinsulinaemia due to the SUR1 R1420H variation suppresses and induces *GCK* and *G6PC2* gene expression, respectively, to regulate insulin secretion in low glucose conditions but the dysregulation of these two genes results in the lower insulin secretory response to high glucose seen in the SUR1 1420RH SC-islets. To test this hypothesis, we have generated isogenic iPSCs with hemizygous deletion of *G6PC2* in isogenic iPSCs heterozygous for the SUR1 R1420H variation. Our ongoing studies include a more detailed assessment of insulin secretion dynamics, glycolytic flux by measuring glycolysis intermediates under different glucose exposure, and calcium influx studies from SC-islets generated using these double-edited cells to understand the role of *G6PC2* in beta cell maturation and its effect on GSIS in the genomic background of the SUR1 R1420H variation.

The dysregulation of *GCK* and *G6PC2* gene expression in 1420RH SC-islets is also consistent with our results that show improved first-phase insulin secretion when the 1420RH SC-islets were treated with the GCK activator dorzagliatin [53, 54]. Under high glucose stimulation, 1420RH SC-islets had improved first-phase insulin secretion when treated with dorzagliatin while there was little improvement during second-phase insulin secretion. This is consistent with a recently proposed compartmentalised model of beta cell metabolism where the K_ATP_ channel closure is initiated by glycolysis mediated ADP lowering; membrane-localised pyruvate kinase driven hydrolysis of phosphoenolpyruvate generates ATP and lowers ADP thereby leading to K_ATP_ channel closure while oxidative phosphorylation helps sustain the membrane depolarisation required for continuous second-phase insulin secretion [55, 56]. This may explain why increasing glycolytic flux in heterozygous 1420RH SC-islets by activating GCK with dorzagliatin only improves the first-phase response.

Single-cell differential gene expression analyses also identified several important genes that were dysregulated in 1420RH and 1420HH SC-alpha cells, including *ID1*, *CRH* and *GCG*. *ID1* and *CRH* were both upregulated in 1420HH and 1420RH SC-alpha cells. In mouse models, *Id1* has been implicated as a negative regulator of insulin secretion and *Id1*^−/−^ mice have improved response to glucose challenge [57]. *CRH* codes for corticotropin-releasing hormone, which can promote beta cell proliferation and regulate insulin secretion by binding to CRH receptors expressed on beta cells [58]. The most intriguing dysregulated gene was *GCG*, the expression of which was directionally consistent and downregulated in both 1420HH and 1420RH SC-alpha cells but upregulated in both SC-beta cells. Studies have reported expression of *GCG* in single-cell sequencing from SC-beta cells and human beta cells [29, 59] and co-expression of insulin and glucagon has been reported in type 2 diabetes [60, 61]. For example, SARS-CoV-2 infection of beta cells, which has been proposed to increase the risk for developing diabetes, induces beta cell transdifferentiation leading to increased expression of alpha cell markers in beta cells including *GCG* [62]. Beta cell dedifferentiation and transdifferentiation have been proposed as important contributors towards beta cell failure in type 2 diabetes [63–65]. Aldehyde dehydrogenase 1A3 (ALDH1A3) is a marker of dedifferentiation [65, 66]; however, we did not observe ALDH1A3 expression in K_ATP_ channel variant SC-islets by immunostaining (data not shown). Therefore, more mechanistic studies are needed to understand the role of increased *GCG* expression in SC-beta cells with the SUR1 R1420H variation. Among the other SC-beta cell dysregulated genes were the three S100 family genes, *S100A13*, *S100A6* and *S100A11*, which are involved in Ca^+^ binding and signalling. These genes were upregulated in both 1420HH and 1420RR SC-beta cells. This is consistent with a previous report showing that S100 genes are upregulated in *ABCC8*^−/−^ mice and with SUR1 LoF variation leading to an increase in Ca^+^ entering the cells [67].

Our study has limitations that could potentially be addressed in the future. First, we used in vitro methods to generate mature SC-islets instead of in vivo maturation achieved by transplanting immature SC-islets into mice. There is a prior report suggesting that in vivo SC-islet maturation in mice leads to a transcriptomic profile more comparable with human pancreatic islets [29]. Second, our study focused on understanding the effect of the SUR1 R1420H variation on insulin secretion from immature and mature SC-islets and identifying the transcriptomic differences in mature SC-islets with the variation. However, we have not carried out assessment of the effect of the variation on SUR1 content, trafficking or turnover, which will be the focus of future studies.

In conclusion, we show that the SUR1 R1420H variation identified in Indigenous Americans leads to insulin hypersecretion in basal condition in both immature and mature SC-islets, suggesting that this is a K_ATP_ channel LoF variation. We further show that, in the homozygous state, this variation leads to a substantial loss of GSIS response, while in the heterozygous state, this variant results in a significantly reduced insulin secretory response that explains the very early onset of diabetes in the one homozygous individual and increased type 2 diabetes risk in the adult heterozygous carriers. We also identified transcriptomic differences in SC-beta cells and SC-alpha cells carrying this variation. This model system and transcriptomic data will be valuable for identifying and screening potential drug therapies for treating hyperinsulinaemia and type 2 diabetes.

## Supplementary Information

Below is the link to the electronic supplementary material.

ESM (PDF 13.2 MB)

ESM Tables (XLSX 1.59 MB)

## Data Availability

Further information about resources and reagents should be directed to the corresponding author. There are restrictions to the availability of human iPSCs lines used and generated in this study because these cell lines were generated from blood cells from Indigenous American individuals. The Indigenous community has not consented to the sharing of biospecimens with outside researchers and collaborators. All other reagents are available on reasonable request. Single-cell sequencing data reported in this study cannot be deposited in a public repository because data were generated from Indigenous American iPSCs. Inquiries about these data can be made to the corresponding author. See the database of Genotypes and Phenotypes (dbGaP; dbgap.ncbi.nlm.nih.gov/home; accession no.: phs002490.v1.p1) for details concerning data requests. All source codes can be found in the GitHub repository under https://github.com/Koushik-Cheranda/SC-islet-scRNAseq-analysis-R-codes.

## Abbreviations

ALDH1A3: Aldehyde dehydrogenase 1A3
CXCR4: C-X-C chemokine receptor type 4
DE: Definitive endoderm
d-GSIS: Dynamic GSIS
GCG: Glucagon
GCK: Glucokinase
GSIS: Glucose-stimulated insulin secretion
HHI: Hyperinsulinaemic hypoglycaemia during infancy
HVG: Highly variable gene
INS: Insulin
iPSC: Induced pluripotent stem cell
IS1: Isogenic cell line 1
IS2: Isogenic cell line 2
K_ATP_: ATP-sensitive potassium (channel)
Kir6.2: Inwardly rectifying potassium channel subunit 6.2
LoF: Loss of function
Log_2_FC: Log_2_ fold change
NBD1: Nucleotide-binding domain 1
NBD2: Nucleotide-binding domain 2
PBMC: Peripheral blood mononuclear cell
PC: Principal component
PP: Pancreatic progenitor
S4D4: Stage 4 day 4
S6D7: Stage 6 day 7
S7W1: Stage 7 week 1
S7W2: Stage 7 week 2
SC-alpha: iPSC-derived alpha cells
SC-beta: iPSC-derived beta cells
SC-delta: iPSC-derived delta cells
SC-islets: iPSC-derived islets
scRNA-seq: Single-cell RNA-seq
sgRNA: Guide RNA
SI: Stimulation index
SSEA4: Stage-specific embryonic antigen 4
SUR1: Sulfonylurea receptor 1
UMAP: Uniform Manifold Approximation and Projection
UMI: Unique molecular identifier
