# Supplementary material for: Modelling the effects of human SUR1 R1420H variation on insulin secretory function using isogenic iPSC-derived pancreatic islets
Source: Diabetologia. 2025 Nov 25;69(2):399–420. doi: 10.1007/s00125-025-06605-1 (PMC12779732; doi:10.1007/s00125-025-06605-1)
Supplement: Supplementary file 1 — ESM (PDF 13.2 MB) [file 125_2025_6605_MOESM1_ESM.pdf]

Modelling the effects of human SUR1 R1420H variation on insulin secretory function using isogenic iPSC-derived pancreatic islets

Anup K. Nair, Katiya Barkho, Koushik Ponnanna Cheranda, Michael Traurig, Jeffrey R. Sutherland, Divya Anup, Clifton Bogardus, Leslie J. Baier

ESM Methods

Human iPSC derivation and culture

Human iPSCs (parental iPSCs) used in this study were derived from peripheral blood mononuclear cells (PBMCs) of Indigenous American individuals who provided informed consent. Human iPSCs used in this study were from female subject (age > 18 years) who were diagnosed with type 2 diabetes. PBMCs were reprogrammed into iPSCs at the NIH-National, Heart, Lung and Blood Institute (NHLBI) iPSC core facility using a Sendai virus-based reprogramming kit (Thermo Fisher, Waltham, MA, USA). The iPSCs were initially maintained in mTeSR1/Matrigel (catalogue no. 85850, Stem Cell Technologies, Vancouver, BC, Canada/catalogue no. 354227, Corning, Corning, NY, USA) culture system at 37°C in humidified 5% CO_2 incubator. Tests for Sendai virus and mycoplasma (catalogue no. 30-1012K, ATCC, Manassas, VA, USA) were routinely performed during passaging. Passaging was done using Versene solution (catalogue no. 15040066, Thermo Fisher). Sendai virus free and mycoplasma negative iPSCs (at passage >10) were adapted (5 passages) and expanded in DEF-CS 500 culture system (catalogue no. Y30010, Takara Bio, San Jose, CA, USA) at 37°C in humidified 5% CO_2 incubator. In this culture system, passaging was performed every third day with TrypLE select 1X (catalogue no. 12563011, Thermo Fisher) when cells reached a dense

confluency. All downstream iPSC culture were done using DEF-CS 500 culture system. After adaptation, iPSCs were characterised for pluripotency, differentiation potential and genomic stability. Large batch of characterised iPSCs with stable karyotype were then cryopreserved. Cells from this batch of iPSCs were used for the generation of isogenic cell lines.

Generation of isogenic iPSCs

Guide RNA (sgRNA) generation, iPSC editing, and single cell cloning was performed using the Cellartis iPSC rCas9 Electroporation and Single-Cell Cloning system (catalogue no. 632643, Takara Bio) following manufacturer’s instructions. A PAM site (TGG) 5bp upstream of the target site was selected for the generation of guide RNA (sgRNA). The 20bp target sequence used was – 5’ *GACGGGGTCCTGCAGGATGA* 3’. A DNA template was first generated by PCR using the scaffold template (included with the kit) and a primer with the target sequence and T7 promoter sequence. The DNA template was then reverse transcribed using T7-polymerase mix (included with the kit) to generate the sgRNA. The sgRNA was subsequently digested with DNase I, purified using the IVT RNA clean-up kit (included with the kit) and quantified. The sgRNA was complexed with the Cas9 recombinant protein and along with an asymmetric donor DNA (ultramer DNA oligo, IDT, Coralville, IW, USA) was used for electroporation. The donor DNA sequence was:

1. To change 1420H to 1420R –
5’TCACTGGGACCCATGCAGGGCACATCATCATTGATGGCATTGACATCGCCAAAC
*TGCCGCTGCACACCCTGCGCTCACGCCTCTC**G**ATCATCCTGCAGGACCCCGTCCT*
CTTCAGCGGCACCATCCG3’

2. To change 1420R to 1420H –

*5’TCACTGGGACCCATGCAGGGCACATCATCATTGATGGCATTGACATCGCCAAACTGCCGCTGCACACCCTGCGCTCACACCTCTC**G**ATCATCCTGCAGGACCCCGTCCTCTTCAGCGGCACCATCCG3’*

The target site is underlined. A C to G change (highlighted in bold) resulting in a synonymous variation was also introduced into the donor DNA to disrupt the PAM site and prevent further cleavage of the corrected edit. Following electroporation (using Neon transfection system, Thermo Fisher) and recovery of edited iPSCs (~4-5 days), single cell cloning was done using limiting dilution (0.5 cells/well of a 96-well plate). All wells were screened under microscope (EVOS XL Core, Thermo Fisher) to identify single colonies, and these colonies were expanded for 10-14 days before further passaging into 48-well plates. The residual cells left in the 96-well plate were used for DNA isolation using QuickExtract DNA extraction solution (catalogue no. QE09050, Lucigen, Middleton, WI, USA). DNA was Sanger sequenced to identify successful edits, and these colonies were further expanded to establish isogenic cell lines. All new cell lines were also Sanger sequenced to confirm the desired edits before further expansion and characterisation of isogenic cell lines with the desired edit. All isogenic iPSC cultures were done using DEF-CS 500 culture system at 37°C in humidified 5% CO_2 incubator.

RNA isolation and quantitative real-time PCR

RNA was isolated using the RNeasy plus micro kit (catalogue no. 74034, Qiagen) and 1-2µg of total RNA was converted to cDNA using the high-capacity RNA-to-cDNA kit (catalogue no. 4387406, Thermo Fisher). Real-time PCR was done using validated gene specific primers listed in ESM Table 3 and PowerUP SYBR green master mix

(catalogue no. A25777, Thermo Fisher). Real-time PCR was primarily used to ensure proper differentiation by assessing the expression of stage specific marker genes. *TBP* was used for normalisation and relative expression was analysed using $2^{-\Delta\Delta C_t}$ method. SDS RQ manager (ver. 1.2.1, Applied Biosystems) was used for the analysis of real-time PCR data. For monitoring differentiation, real-time PCR data from all stages of differentiation were analysed together and expression from one of the 1420RR undifferentiated iPSCs was used as a reference to calculate relative expression in all other samples from different stages of differentiation. This stage specific relative expression was used for statistical comparison between 1420RR vs 1420RH and 1420RR vs 1420HH cells. Alternatively, stage specific difference in expression in 1420RH and 1420HH cells were also compared using expression from one of the 1420RR cells from the same stage. For monitoring SC-islet maturation, fold change in expression of marker genes during day 27 (S7W1) and day 34 (S7W2) were calculated using expression in one of the 1420RR day 20 (S6D7) cells. The fold changes were then compared between 1420RR vs 1420RH and 1420RR vs 1420HH.

Flow cytometry

For surface antigen staining (TRA-1-60, Stage-Specific Embryonic Antigen 4 [SSEA4], C-X-C chemokine receptor type 4 [CXCR4] and cluster of differentiation 117 [CD117]), cells were harvested using TrypLE select 1X (Thermo Fisher), washed twice with dPBS and resuspended in stain buffer (catalogue no. 554656, BD Biosciences, Franklin Lakes, NJ, USA). One million cells were then incubated with fluorophore conjugated primary antibodies or isotype control antibodies for 30 minutes at room temperature. Cells were washed twice with stain buffer and resuspended in 500 µL of stain buffer and

filtered using Flowmi cell strainers (catalogue no. H13680-0070, SP Bel-Art, Wayne, NJ, USA) before data capture. For intracellular antigen staining (pancreatic and duodenal homeobox 1 [PDX1], NK6 homeobox 1 [NKX6-–1], insulin [INS] and glucagon [GCG]), cells were harvested using TrypLE select 1X and washed once with ice cold dPBS before fixing with 4% paraformaldehyde (catalogue no. 28908, Thermo Fisher) for 30 minutes on ice. Fixed cells were washed once with ice cold dPBS and incubated in blocking and permeabilization buffer (PBS + 0.1% Triton X-100 + 5% donkey serum) for 1 hour at 4°C. The blocking buffer was removed by centrifugation and the cells were resuspended in appropriate primary antibodies or isotype controls diluted in blocking buffer and incubated overnight at 4°C. Cells were then washed twice and incubated in fluorophore conjugated secondary antibodies diluted in blocking buffer and incubated for 2 hours at 4°C. Cells were washed three times with sorting buffer (PBS + 0.5% BSA) and resuspended in 500µl sorting buffer before filtering using Flowmi cell strainers. Data was captured using an Accuri C6 plus flow cytometer (BD Biosciences) and analysed using FlowJo software v 10.6.1. Isotype control antibodies were used to determine gating.

Dynamic GSIS

Perifusion system V5 (Biorep, Miami, FL, USA) was set up per manufacturer's instructions. Handpicked, size matched immature SC-islets (~100) and mature SC-islets (~70) were sandwiched in Bio-Gel P-4 polyacrylamide beads (catalogue no. 1504124, Bio-Rad, Hercules, CA, USA) in a perifusion chamber and perifused with KRB buffer (128 mmol/l NaCl + 5 mmol/l KCl + 2.7 mmol/l $CaCl_2$ + 1.2 mmol/l $MgSO_4$ + 1 mmol/l Na_2HPO_4 + 1.2 mmol/l KH_2PO_4 + 5 mmol/l $NaHCO_3$ + 10 mmol/l HEPES + 0.1% BSA,

gassed with 95%/5% O_2/CO_2 mix and pH adjusted to 7.4 with NaOH) containing 2mM glucose for equilibration (90 minutes). This was followed by perifusion with KRB buffer containing different secretagogues as indicated in figure legends at a flowrate of 100ul/min. The effluent was collected every minute and assessed for secreted insulin using human insulin ELISA kit (catalogue no. 10-1113-01, Mercodia, Winston Salem, NC, USA). The entire perifusion run was carried out at 37°C and the samples were collected at 4°C using the in-built system. SC-islets were collected from chambers at the end of the perifusion to quantify total insulin and total DNA (Quant-iT PicoGreen dsDNA Assay Kits, catalogue no. P7589, Thermo Fisher).

Immunocytochemistry

For immunostaining of SC-islets, cells were dissociated into single cells with TrypLE select 1X (Thermo Fisher). A monolayer of cells was then deposited on Shandon cytoslides (catalogue no. 5991056, Fisher Scientific) using a cytospin 4 centrifuge (catalogue no. A78300003, Fisher Scientific). Cells were fixed using 4% paraformaldehyde (Thermo Fisher) for 20 minutes at room temperature. Fixed cells were washed twice with wash buffer (PBS + 0.1% BSA) and incubated in blocking buffer (PBS + 0.3% triton-x-100 + 10% donkey serum) for 45 minutes at room temperature for blocking and permeabilization. Cells were then incubated overnight at 4°C in diluted primary antibody (dilution buffer - PBS + 1% BSA + 0.3% triton-x-100, 0.01% sodium azide + 1% donkey serum). The next day, cells were washed three times with wash buffer and incubated for 1 hour protected from light in appropriate secondary antibody diluted in dilution buffer. After incubation, the cells were again washed thrice with wash buffer and counterstained using NucBlue Fixed Cell ReadyProbes (catalogue no.

R37606, Thermo Fisher) and mounted using ProLong Diamond Antifade Mountant (catalogue no. P36961, Thermo Fisher) before imaging. Images were acquired using an EVOS FL cell imaging system (catalogue no. AMF4300, Thermo Fisher) with built in image acquisition software.

Hormone content

Mature SC-islets (~50) were collected in ice-cold Tris-EDTA (pH 7.4) and briefly sonicated to disrupt cell membrane. Cell debris was removed by centrifugation and aliquots of supernatant were used for measuring insulin (catalogue no. 10-1113-01, Mercodia), proinsulin (catalogue no. 10-1118-01, Mercodia), glucagon (catalogue no. 10-1271-01, Mercodia) and DNA (Quant-iT PicoGreen dsDNA Assay Kit).

Single-cell RNA library preparation

Since 1420RH SC-islets only had mild basal hyperinsulinemia, we decided to maintain the mature SC-islets (S7W2) in culture for an additional 2 weeks (S7W4) before using them for studying transcriptomic differences. S7W4 (Day 48) SC-islets were generated using four independent differentiations using nine IS2 cell lines. Three cell lines, one for each genotype, were used in two independent differentiations (IS2-RR2, IS2-RH2 and IS2-HH2). We monitored efficiency during differentiation by flow cytometry staining of PP markers PDX1 and NKX6-1 during stage 4 and generation of SC-beta and SC-alpha cells were confirmed by immunofluorescence staining of S7W2 islets for INS, GCG and NKX6-1 (see ESM Figure 12). SC-islets were dissociated into single-cell suspensions using TrypLe select 1X (Thermo Fisher) and single-cell libraries were prepared following the 10X Genomics Single Cell 3’ v3 Reagent Kit and protocol per manufacturer’s

instructions (Chromium Single Cell 3' GEM, Library & Gel Bead Kit v3, Chromium Single Cell B Chip Kit and Chromium i7 Multiplex Kit, catalogue no. 1000092, 1000073 and 120262 respectively, 10X Genomics, Pleasanton, CA). Libraries were sequenced using an Illumina NovaSeq 6000 System at SeqMatic (Fremont, CA) at recommended read setting: read 1, 26 bps; read 2, 91 bps; I7 index, 8 bps.

Secondary structure prediction using AlphaFold 3 and SWISS-MODEL

AlphaFold3 (v3.0.1) and SWISS-MODEL with default parameters was used for structure prediction of the SUR1 monomer. Subsequent molecular docking of Mg-ADP and Mg-ATP to the predicted protein monomer was carried out using AutoDock Vina (v1.2.5) docking engine. The Simplified Molecular Input Line Entry System (SMILES) for MgADP and MgATP were retrieved from the PubChem database. The binding efficiency for MgADP was −5.585 kcal/mol for 1420R SUR1 and −5.345 kcal/mol for 1420H SUR1, while for MgATP, it was −5.828 kcal/mol for 1420R SUR1 and −5.617 kcal/mol for 1420H SUR1. These results indicate a change in binding affinities between 1420R and 1420H SUR1 for MgADP and MgATP.

<u>SMILES</u>

Mg-ATP

C1=NC(=C2C(=N1)N(C=N2)[C@H]3[C@@H]([C@@H]([C@H](O3)COP(=O)(O)OP(=O)([O-])OP(=O)(O)[O-])O)O)N.[Mg+2]

or

[Mg].Nc1ncnc2n(cnc12)C3OC(COP(O)(=O)OP(O)(=O)OP(O)(O)=O)C(O)C3O

Mg-ADP

[Mg++].NC1=C2N=CN(C3OC(COP([O-])(=O)OP(O)([O-])=O)C(O)C3O)C2=NC=N1

ESM Tables

ESM Table 3: Primers used for real-time PCR

Gene	Forward primer	Reverse primer
ABCC8	CTGCTGTCCAAAGGCACCTA	CTGAATGTCCTTCCGCACCT
ARX	AGGACAGCGTGTGCCTCTC	CCACTTGGCCCGACGGTTC
CHGA	AAC ACAGCGGTTTTGAAGATGA ACT	CTCCATAACATCCTTGGATGATGGCTCT
FOXA2	TCCGACTGGAGCAGCTACTATG	CCACGTACGACGACATGTTC
GCG	ACGTTCCCTTCAAGACACAG	AGGATCACTGAGTGGGTCTG
GCK	GTGTACAAGCTGCACCCCA	TCCGACTCGATGAAGGTGAT
GLP1R	GAGGTCATCTTTGCCTTTGTG	TAATATGGCCACCATCAGCC
INS	ACACCTGGTGGAAGCTCTCT	GGGTCTTGGGTGTGTAGAAG
KCNJ11	CCTGTGTCACCAGCATCCACT	CGTTGATCATGAGCCCCACGA
MAFB	TCAAGTTCGACGTGAAGAAGG	GTTCATCTGCTGGTAGTTGCT
NEUROD1	CCCTGTACACCCCTACTCCT	GAGGCTTAACGTGGAAGACA
NEUROG3	TCCCTCTACTCCCCAGTCTC	AGAAAATCTGAGAAAGCCAGAC
NKX2-2	GGCCTTCAGTACTCCCTGCA	GGGACTTGGAGCTTGAGTCCT
NKX6-1	CACACGAGACCCACTTTTTC	CCGCCAAGTATTTTGTTTGT
POU5F1	AGTGAGAGGCAACCTGGAGA	ACACTCGGACCACATCCTTC
PCSK1	ATATTCCCGAAG AGGAGACCTTCA	GCCATTAGGAGATGTATCCCGTTCT
PCSK2	CCAAC TATAATGCCGAAGCAAGT	CCGTGGCTGTTAAACCAGT CA
PDX1	CTTTCCCATGGATGAAGTCTAC	ATAGGAACTCCTTCTCCAGCTC
SLC2A2	TGCATTCAGCAATTGGACC	AGCACTCCAGCAAAGAGGAA
SST	CAGCTCAAGCCTCATTTCAT	CTGTCTGAACCCAACCAGAC
UCN3	GAGGGAAGTCCACTCTCGG	TGTAGAACTTGTGGGGGAGG
TBP	TATAATCCCAAGCGGTTTGC	GCACACCATTTTCCCAGAAC

ESM Table 4: Antibodies and isotype controls

Antibodies	Company	RRID/Catalogue
Mouse anti-Human SSEA-4, IgG3, K, PE conjugated (20ul/test)	BD Biosciences	RRID: AB_1645533 Cat# 560128
Mouse anti-human TRA-1-60, IgM, K, FITC conjugated (20ul/test)	BD Biosciences	RRID: AB_1645492 Cat# 560380
Mouse anti-human CD184, IgG2A, K, APC conjugated (20ul/test)	BD Biosciences	RRID: AB_398616 Cat# 555976
Mouse anti-human CD117, IgG1, K, PE conjugated (2.5ul/test)	BD Biosciences	RRID: AB_10896487 Cat# 561682
Homeobox protein Nkx-6.1; Nkx6.1 antibody - Madsen, O.D.; Hagedorn Research Institute, unconjugated (1:1500)	DSHB	RRID: AB_532378 Cat# F55A10
Insulin (proinsulin; C-peptide) antibody - Madsen, O.D.; Hagedorn Research Institute, unconjugated (1:100)	DSHB	RRID: AB_2255626 Cat# GN-ID4
Goat anti-human PDX-1/IPF1 Antibody, unconjugated (1:400)	R and D systems	RRID: AB_355257 Cat# AF-2419
Mouse anti-human Glucagon Antibody, unconjugated (1:400)	R and D systems	RRID: AB_2107340 Cat# MAB1249
Alexa Fluor™ 488 donkey anti-goat secondary antibody (1:200)	Jackson Immuno Research Labs	RRID: AB_2340430 Cat# 705-546-147
Alexa Fluor™ 488 donkey anti-rat secondary antibody (1:500)	Thermo Fisher	RRID: AB_2535794 Cat# A-21208
Alexa Fluor™ 647 donkey anti-mouse secondary antibody (1:600)	Thermo Fisher	RRID: AB_162542 Cat# A-31571
Mouse IgM Kappa, FITC conjugated, isotype control	BD Biosciences	RRID: AB_395959 Cat# 555583
Mouse IgG3 K, PE conjugated, isotype control	BD Biosciences	RRID: AB_10050453 Cat# 559926
Mouse IgG2A K, APC conjugated, isotype control	BD Biosciences	RRID: AB_398604 Cat# 555576
Mouse IgG1, K, PE conjugated, isotype control	BD Biosciences	RRID: AB_395506 Cat# 554680
Mouse IgG2A isotype control, unconjugated	R and D systems	RRID: AB_357345 Cat# MAB003
Normal Goat IgG, isotype control, unconjugated	R and D systems	RRID: AB_354267 Cat# AB-108-C
Rat IgG2a, isotype control, unconjugated	Thermo Fisher	RRID: AB_2532970 Cat# 02-9688
ChromPure Mouse IgG, isotype control, unconjugated	Jackson Laboratories	RRID: AB_2337188 Cat# 015-000-003

ESM Fig. 1: Characterisation of parent iPSCs

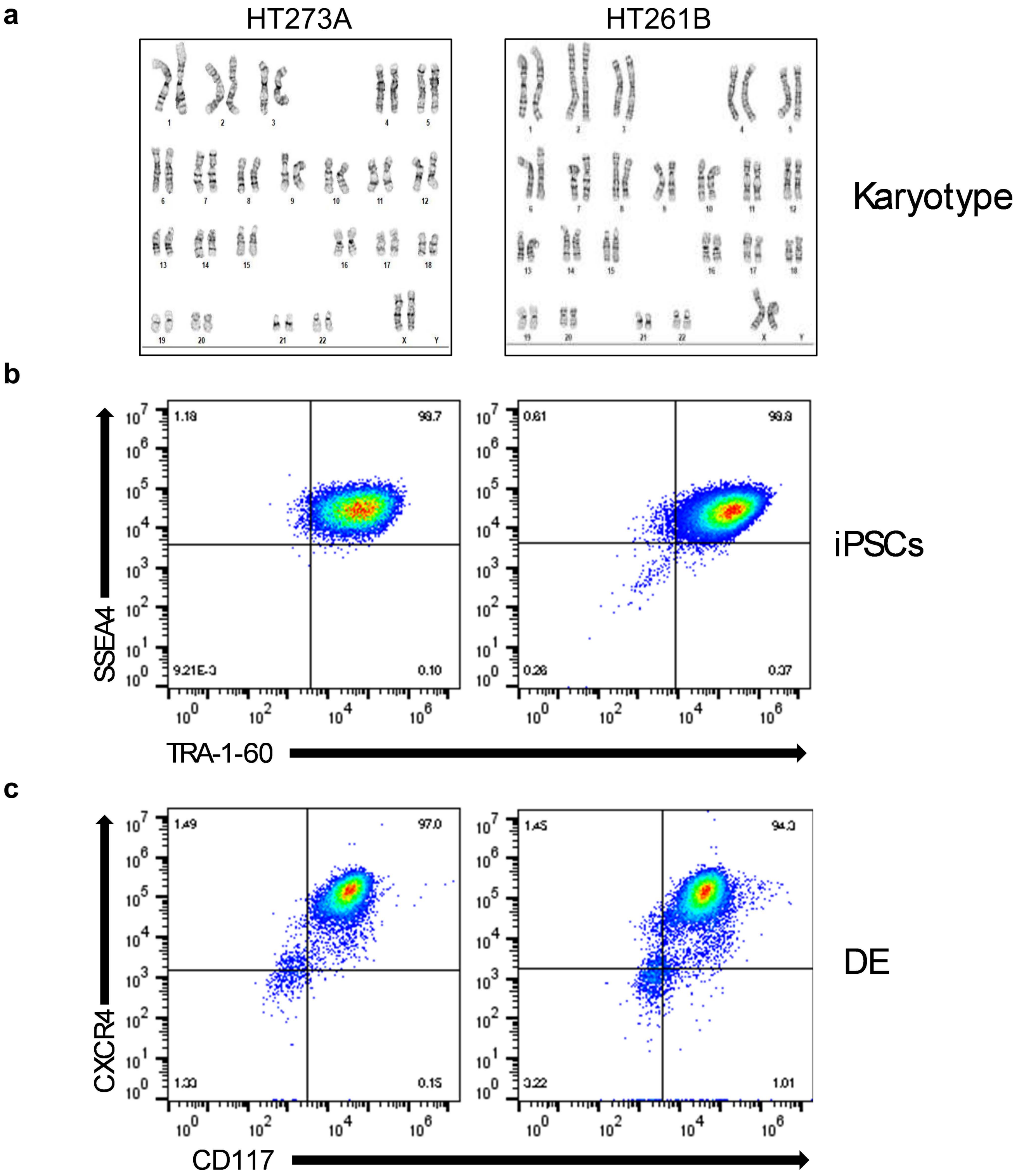

Characterisation of parent iPSCs. a. Karyotype **b.** Flow cytometry analysis of iPSCs by staining for pluripotency markers (representative figure from 2 different flow cytometry experiments using cells from different passage) and **c.** Flow cytometry staining for definitive endoderm (DE) markers after two days of directed differentiation (*n*=1).

ESM Fig. 2: CRISPR design and karyotyping of isogenic cell lines

a

```
          T   L   R   S   R   L   S   I   I   L
5'CACACCCTGCGCTCACGCCTCTCCATCATCCTGCAGGACCCCGTC 3'  Target region
                         CCATCATCCTGCAGGACCCCGTC    Guide RNA
5'..CACCCTGCGCTCACGCCTCTCGATCATCCTGCAGGACCCCG.. 3'  Donor 1
5'..CACCCTGCGCTCACACCTCTCGATCATCCTGCAGGACCCCG.. 3'  Donor 2
```

b

Alignment	Distance (bp)	position	Gene
||||||||[||||||||||||]PAM	0	Exonic	*ABCC8*
--|--|||[||||||||||||]PAM	18496	Intronic	*NRG3*
|-|||--|[||||||||||||]PAM	169	Intronic	*SAMM50*
|-||-||-[||||||||||||]PAM	91822	Intergenic	*ODF2L*
-||-||||[-|||||||||||]PAM	4777	Intronic	*RPL31P7*
|-|--||-[||||||||||||]PAM	0	Exonic	*PIGR*
--|-||||[|-||||||||||]PAM	1067	Intergenic	*KDM5C*
|--|||--[||||||||||||]PAM	2284	Intronic	*FBXL16*
|||--|--[||||||||||||]PAM	7811	Intergenic	*ENPP2*
---|||||[|||-||||||||]PAM	30814	Intergenic	*LL22NC03-121E8.4*
||-|--||[|-||||||||||]PAM	0	Exonic	*MORN1*
|-|-||||[||||-|||||||]PAM	33	Intronic	*CACNA1I*
|-||--||[||-|||||||||]PAM	0	Exonic	*ADCK5*
-|-||||-[||-|||||||||]PAM	3919	Intergenic	*KLF15*
|||||||-[||||-|||||||]PAM	986	Intergenic	*TFF2*
---|||||[||||-|||||||]PAM	4643	Intronic	*RP11-436K8.1*

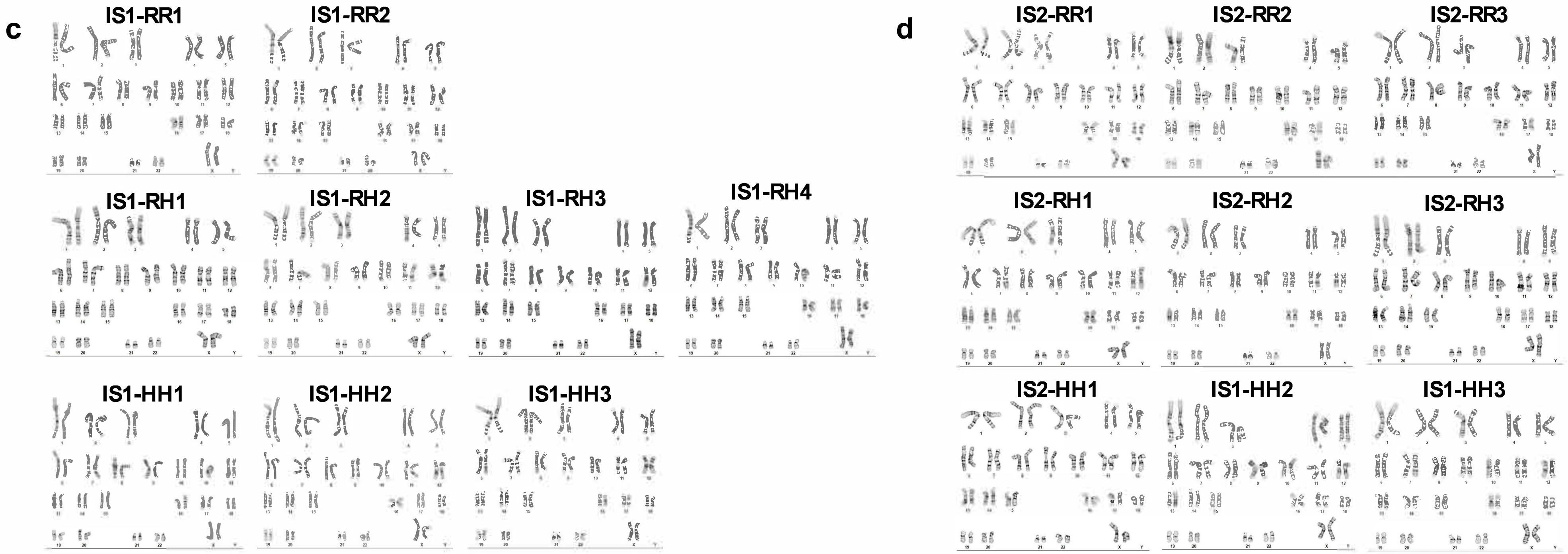

CRISPR design and karyotyping of isogenic cell lines a. CRISPR design. Highlighted in green: PAM site, red: target site, yellow: sgRNA and purple: synonymous change introduced in the PAM site **b.** Top 15 off-target sites. **c-d.** Karyotype of IS1 isogenic cells lines **(c)** and IS2 isogenic cell lines **(d).** For karyotyping 20 metaphase cells were counted, 8 were analyzed and 4 had karyograms to screen for chromosomal abnormalities.

ESM Fig. 3: Chracaterisation of isogenic cell lines.

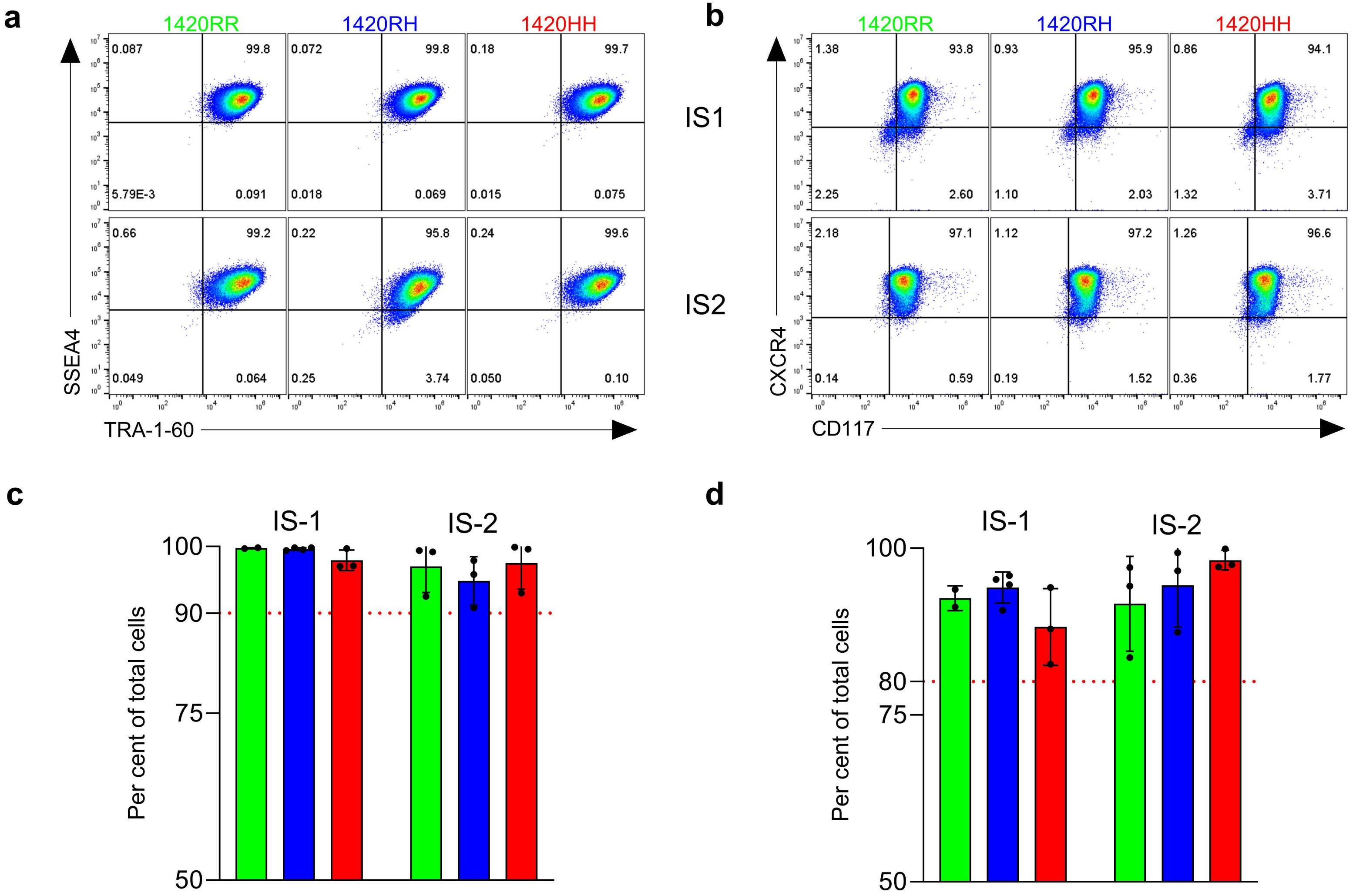

Characterisation of isogenic cell lines a-b. Representative flow cytometry plots for (a) TRA-1-60/SSEA4 staining in IS1 iPSCs (top row) and IS2 iPSCs (bottom row) and (b) CXCR4/CD117 staining in definitive endoderm (DE) cells (S1D2, day 2) generated from IS1 iPSCs (top row) and IS2 iPSCs (bottom row). **c-d.** Flow cytometry results for the 18 selected isogenic iPSCs for (c) pluripotency markers (TRA-1-60 and SSEA4) and (d) DE markers (CXCR4 and CD117). Error bars show mean ± SD.

ESM Fig. 4: Real-time PCR quantification of islet development marker genes in S4D4 cells relative to iPSCs

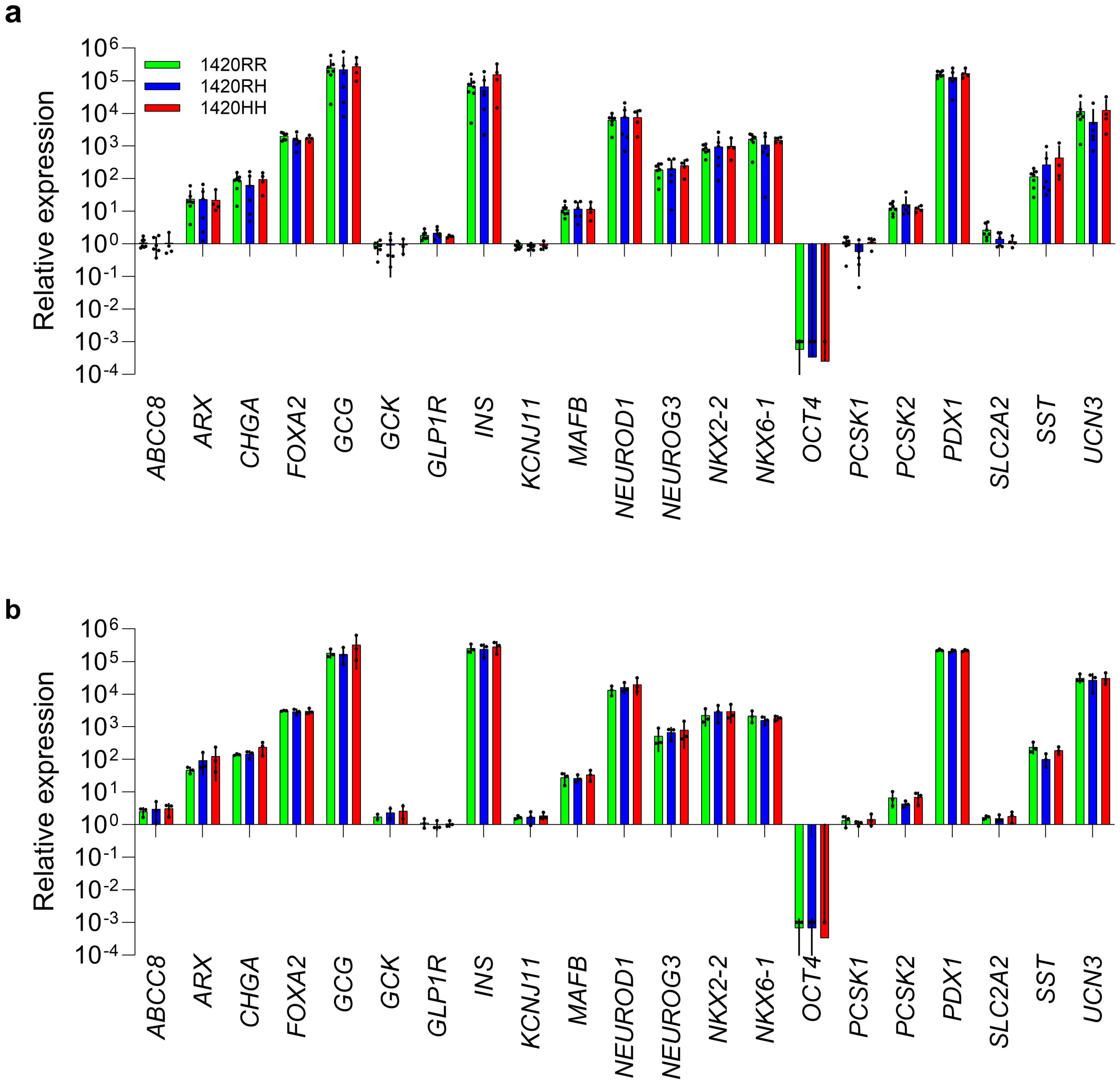

Real-time PCR quantification of islet marker genes in S4D4 cells relative to iPSCs. a-b. Increase/decrease in gene expression relative to iPSCs in S4D4 (PP) cells generated from **a.** IS1 cell lines and **b.** IS2 cell lines. IS1 (5 differentiations): green bars: 1420RR (*n*=7), blue bars: 1420RH (*n*=6) and red bars: 1420HH (*n*=4). IS2 (3 differentiations): *n*=3 for all three genotypes. No significant difference in increase/decrease was observed by genotype. Data shown as mean and SD.

ESM Fig. 5: Real-time PCR quantification of islet devlopment marker genes in S6D7 (day 20) cells relative to iPSCs

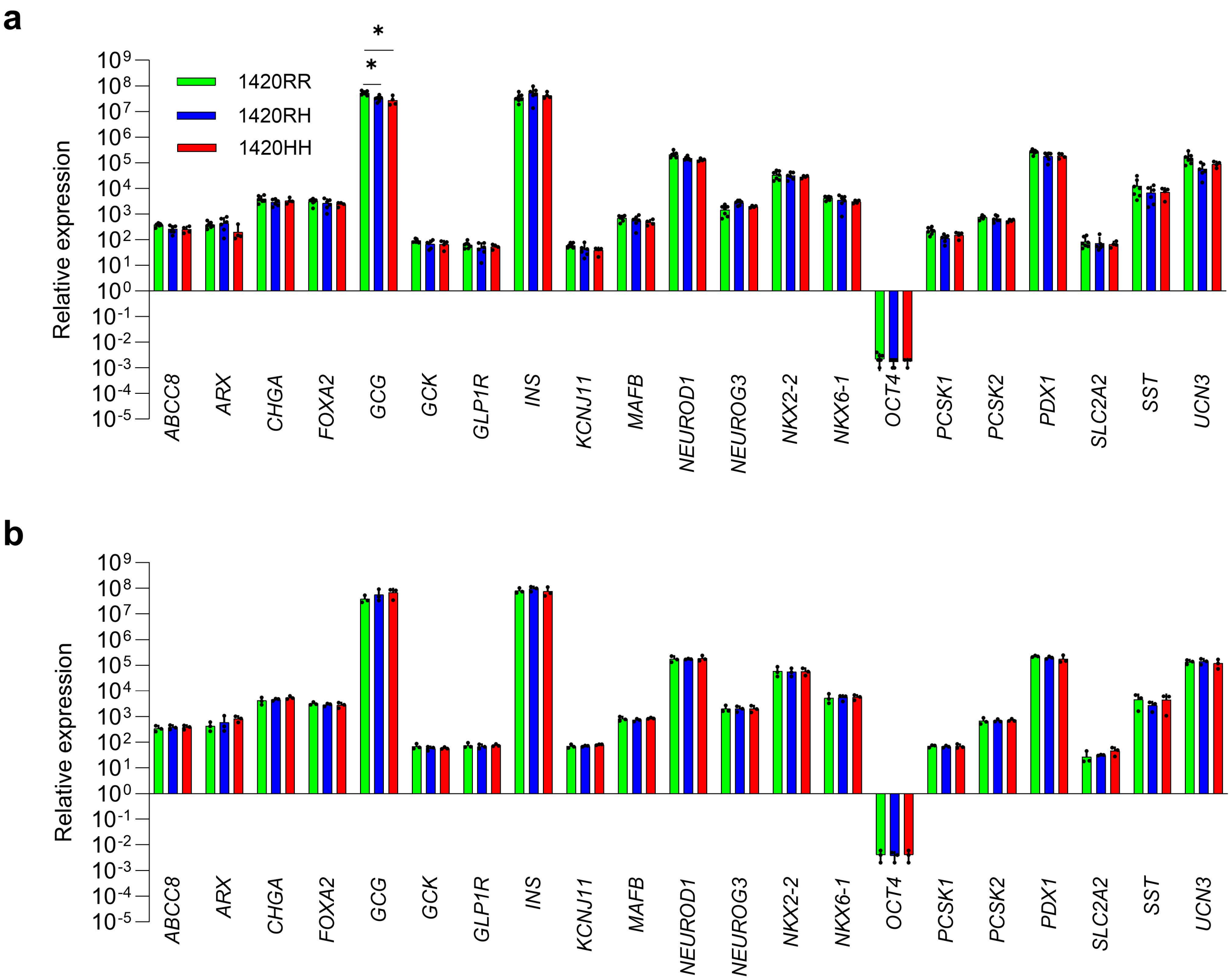

Real-time PCR quantification of islet marker genes in S6D7 cells relative to iPSCs. a-b. Increase/decrease in gene expression relative to iPSCs in S6D7 (immature SC-islets) cells generated from **a.** IS1 cell lines and **b.** IS2 cell lines. IS1 (5 differentiations): green bars: 1420RR (*n*=7), blue bars: 1420RH (*n*=6) and red bars: 1420HH (*n*=4). IS2 (3 differentiations): *n*=3 for all three genotypes. *P<0.05. Datashown as mean and SD.

ESM Fig 6: Expression of select marker genes in 1420RH and 1420HH PP cells and immature SC-islets compared to 1420RR cells

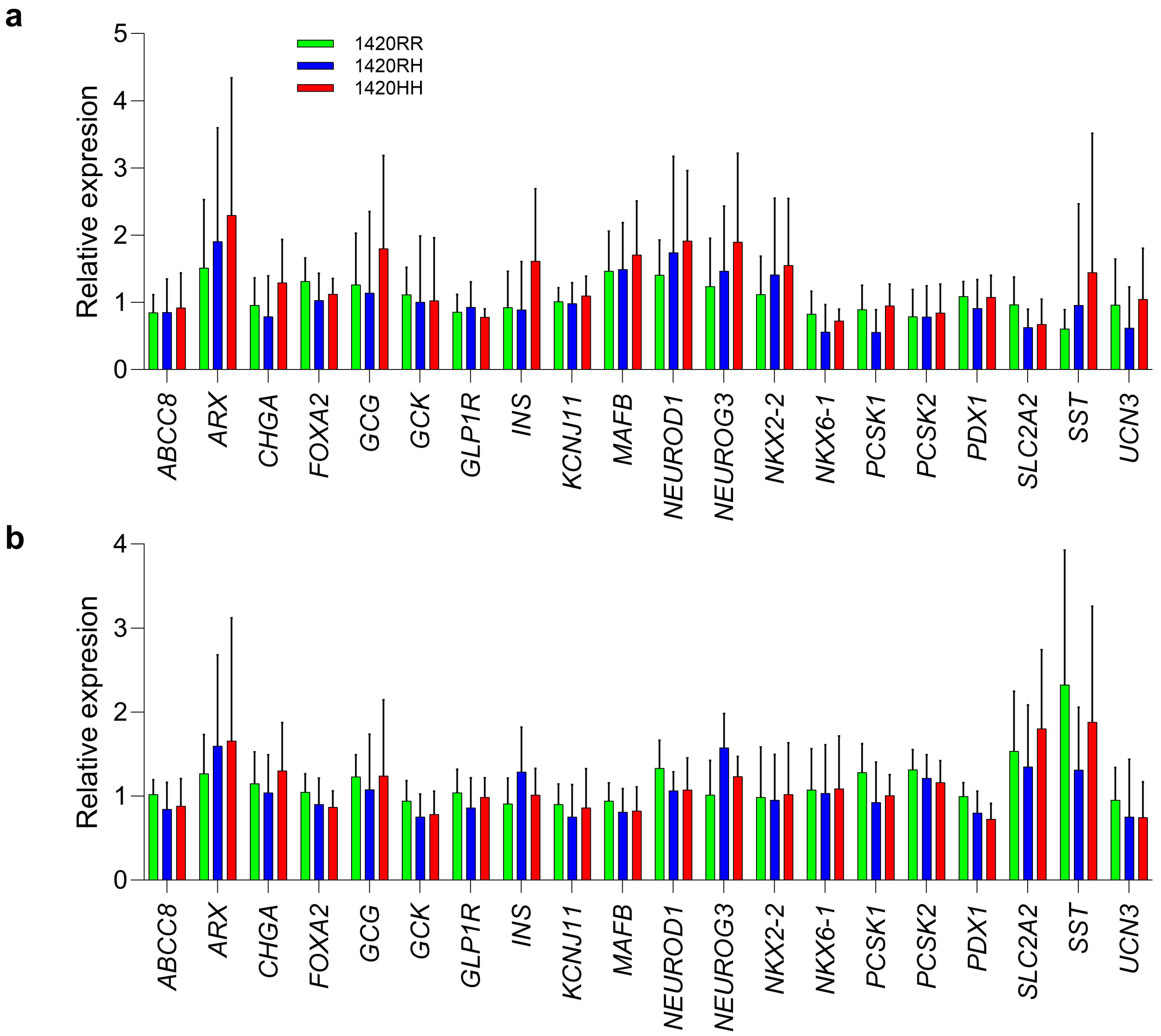

Relative gene expression in 1420RH and 1420HH cells in **a.** PP cells (day 10) and **b.** immature SC-islets (day 20) compared to expression in 1420RR cells. Data from both IS1 cells and IS2 cells were combined for graphical representation. Data from 1420RR cells from one differentiation was used as the calibarator sample to calculate relative expression. No significant difference (after correction for multiple testing using Sidak-Bonferroni method) was seen in gene expression when data from IS1 and IS2 were analyzed seperately or after combination. Data from 8 independent differentiations. 1420RR: *n*=10, 1420RH: *n*=9 and 1420HH: *n*=7. Data represented as mean and SD.

ESM Fig. 7: Immature SUR1 1420HH and 1420RH SC-islets have higher insulin secretion in low glucose condition and are responsive to diazoxide

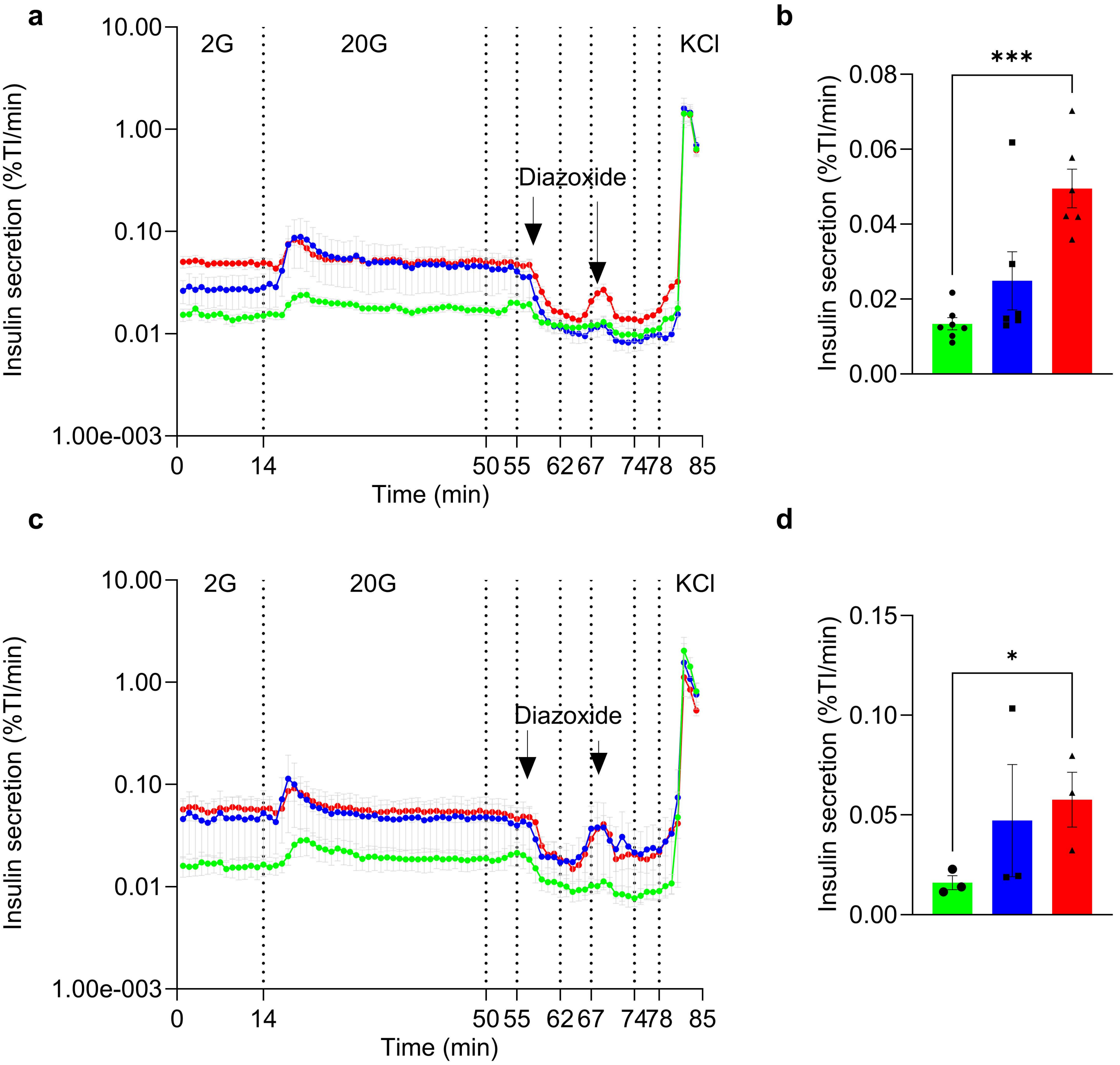

Immature SUR1 1420HH and 1420RH SC-islets have higher insulin secretion in low glucose condition and are responsive to diazoxide. a and c. Insulin secretion as a per cent of total insulin from immature (S6D7) SC-islets generated from a. IS1 cell lines and c. IS2 cell lines during perifusion with 2mmol/l glucose (minutes 1 -14), 20mmol/l glucose (minutes 15-50), diazoxide (minutes 56-62 and 68-74) in the presence of either 2mmol/l glucose or 20mmol/l glucose respectively and KCl (30mmol/l) (minutes 79-85). IS1(4 differentiations), 1420RR, green line (*n*=6); 1420RH, blue line (*n*=5); 1420HH, red line (*n*=6). IS2: (3 differentiations), *n*=3 for all three genotypes. **b and d.** Average insulin secretion (% of total insulin) during the first 12 minutes (2mmol/l glucose stimulation) of perifusion from (b) IS1 immature SC-islets and (d) IS2 immature SC-islets. *p<0.01, ***p<0.001. *n*=same as a and c. Data shown as mean and SEM.

ESM Fig. 8: Real-time PCR analysis of select marker genes in 1420RR, 1420RH and 1420HH SC-islets during S7W1 and S7W2.

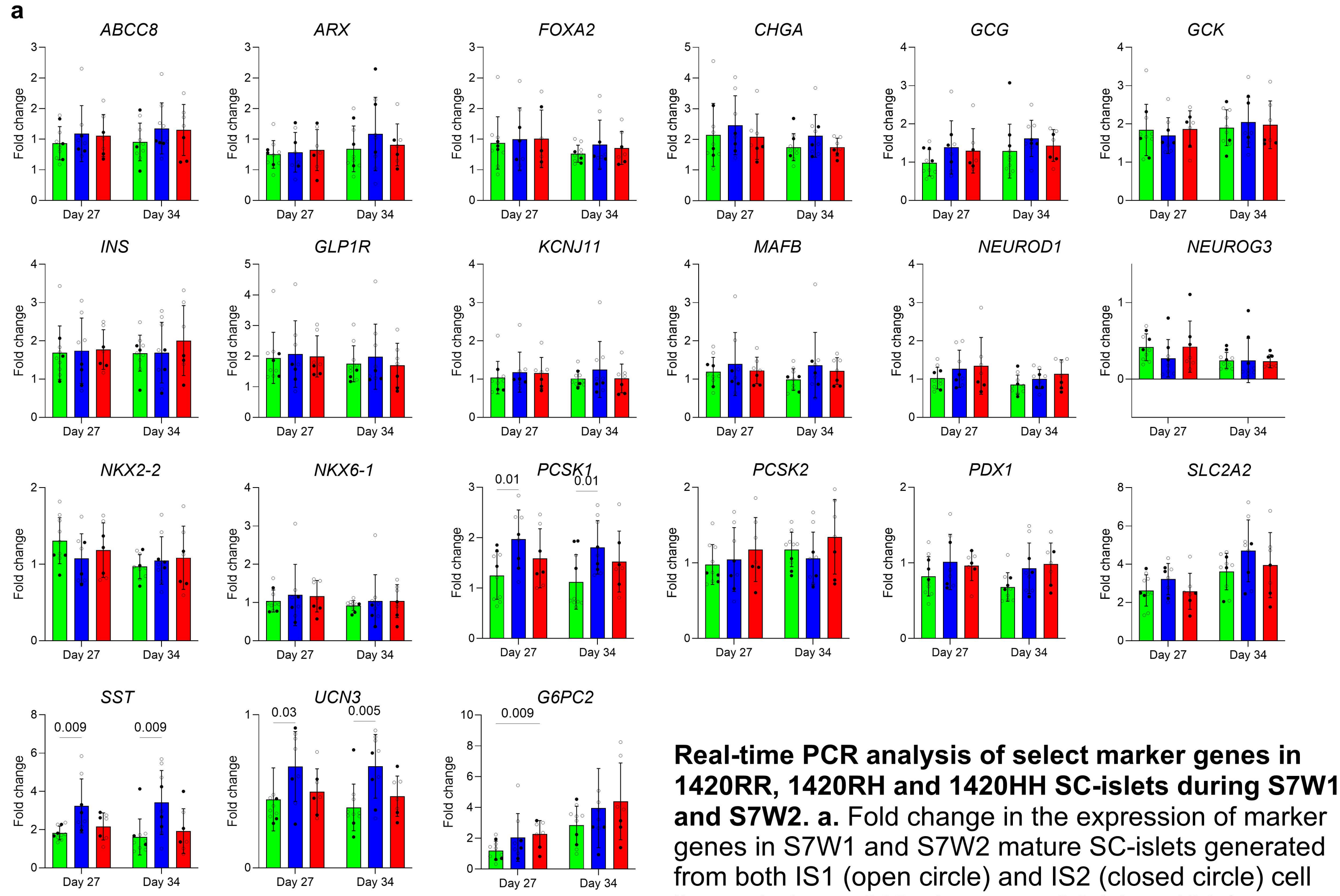

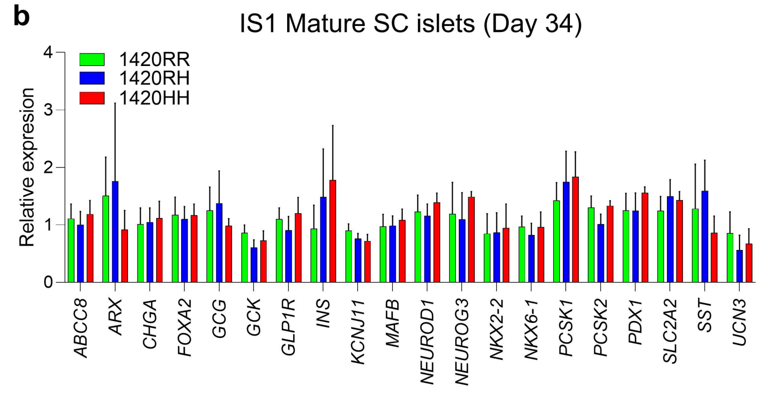

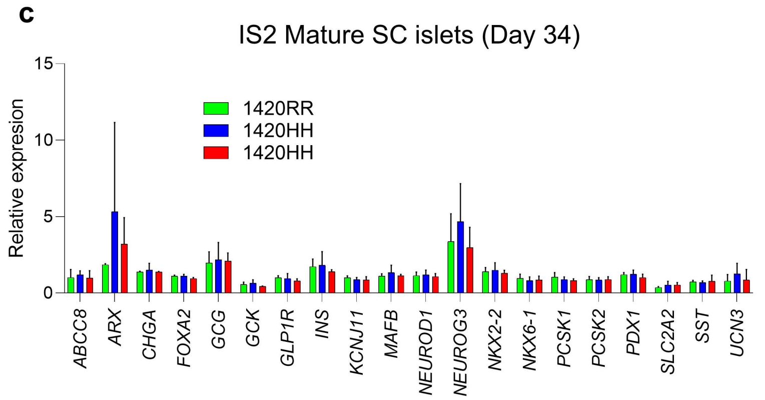

Real-time PCR analysis of select marker genes in 1420RR, 1420RH and 1420HH SC-islets during S7W1 and S7W2. a. Fold change in the expression of marker genes in S7W1 and S7W2 mature SC-islets generated from both IS1 (open circle) and IS2 (closed circle) cell lines relative to S6D7 immature SC-islets. IS1 (5 differentiations): green bars: 1420RR (*n*=7), blue bars: 1420RH (*n*=6) and red bars: 1420HH (*n*=4). IS2 (3 differentiations): *n*=3 for all three genotypes. Listed p values are not adjusted for multiple comparison and were not significant after adjustment. **b-c**. Relative expression of select marker genes in 1420RH and 1420HH SC-islets (day 34) in b. IS1 SC-islets and c. IS2 SC-islets compared to expression in 1420RR SC-islets. No statistically significant difference in expression of these marker gene were seen after correction for multiple testing (Sidak-Bonferroni method). Data from IS1 1420RR mature SC-islets from one differentiation was used as the calibarator sample to quantify relative expression. *n* same as ESM Fig. 8a (above). Data shown as mean and SD.

ESM Fig. 9: No difference in composition between 1420RR, 1420RH and 1420HH SC-islets

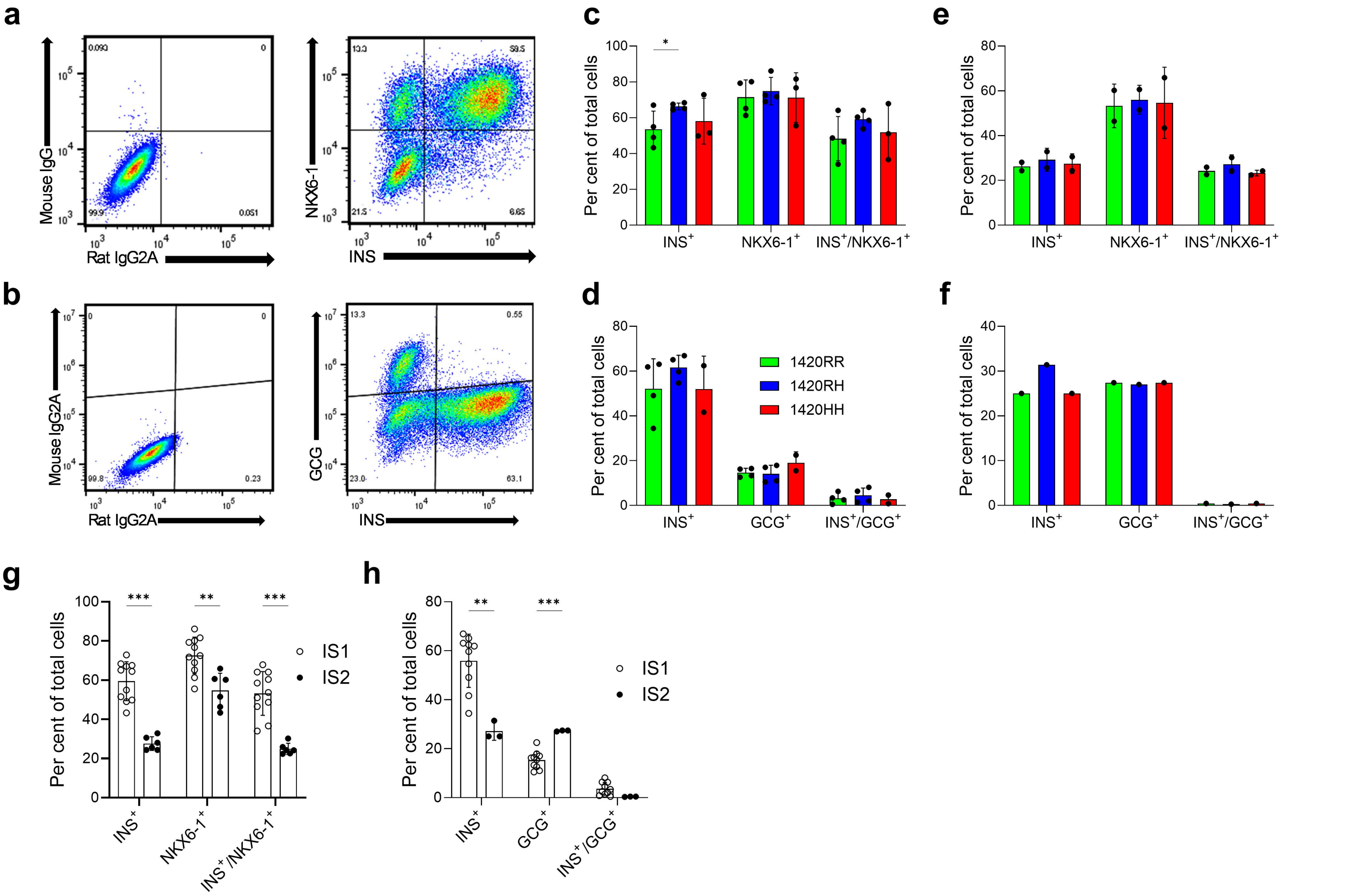

SC-islet composition. a-b. Representative isotype control and antibody staining for (a) INS/NKX6-1 and (b) INS/GCG in mature SC-islets. **c-d.** Flow cytometry results of mature IS1 SC-islets stained for (c) INS and NKX6-1 (1420RR, *n*=4, 1420RH, *n*=4 and 1420HH, *n*=3) and (d) INS and GCG (1420RR, *n*=4, 1420RH, *n*=3 and 1420HH, *n*=2). Data from 3 independent differentiations. **e-f.** Flow cytometry results of mature IS2 SC-islets stained for (e) INS and NKX6-1(*n*=2 for all three genotype). Data from 2 independent differentiations and (f) INS and GCG (data from 1 differentiation). **g-h.** Difference in (g) INS^+ and $NKX6\text{-}1^+$ cells (IS1, *n*=11 and IS2, *n*=6) and (h) INS^+ and GCG^+ cells (IS1, *n*=10 and IS2, *n*=3) between IS1 and IS2 mature SC-islets. $^{**}p < 0.01$, $^{***}p < 0.001$. Data shown as mean and SD.

ESM Fig. 10: Glucose responsive insulin secretion from mature SC-islets with the SUR1 R1420H variation

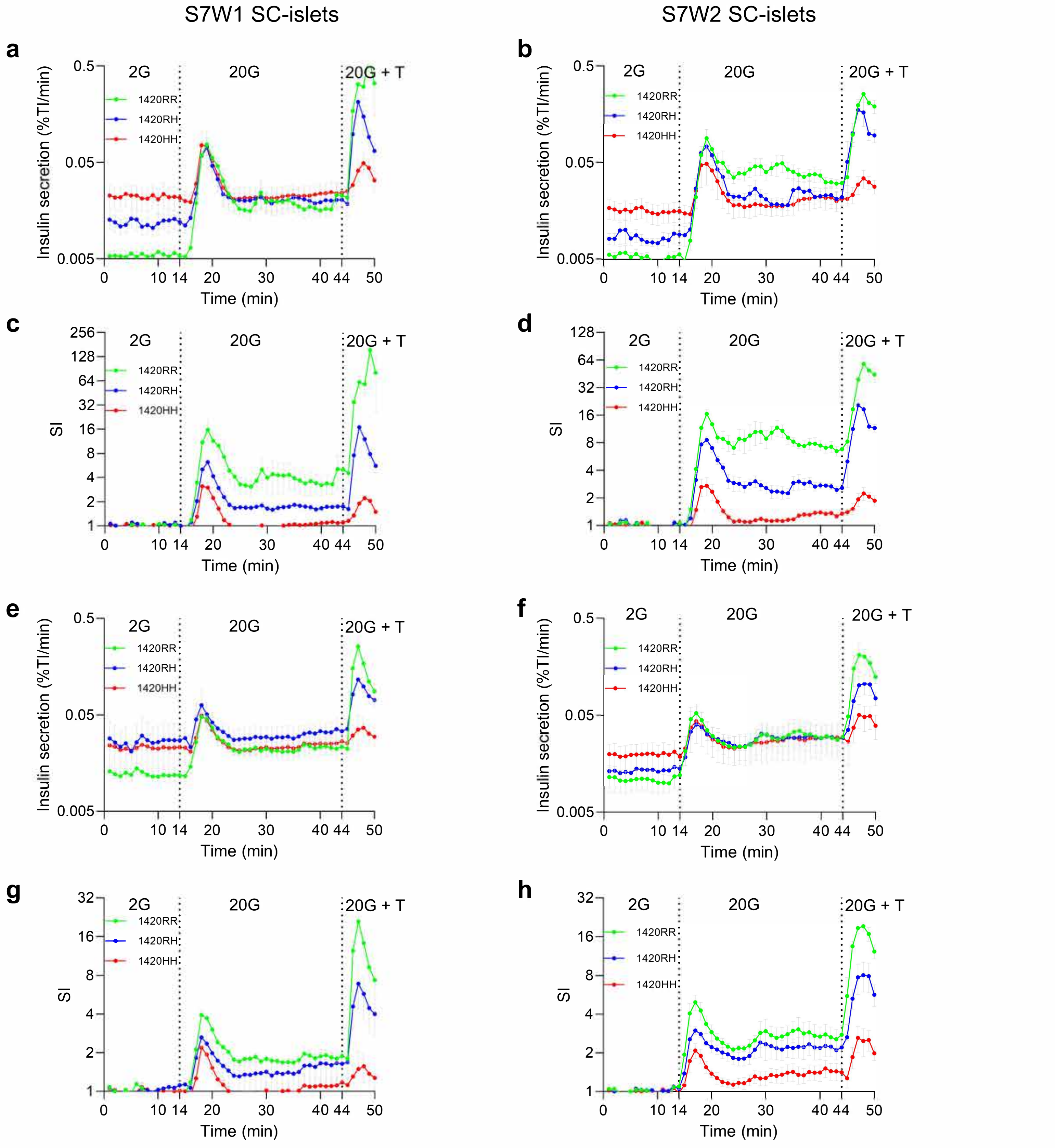

Glucose responsive insulin secretion from mature SC-islets with SUR1 R1420H variation. Insulin secretion during d-GSIS assay shown as a percent of total insulin (a, b, e and f) or stimulation index (SI, c, d, g and h) from mature S7W1 IS1 (a and c), S7W2 IS1 (b and d), S7W1 IS2 (e and g) and S7W2 IS2 (f and h) SC-islets. IS1-S7W1 (4 differentiations): 1420RR, *n*=5; 1420RH, *n*=5; 1420HH, *n*=4. IS1-S7W2 (4 differentiations): 1420RR, *n*=6; 1420RH, *n*=5; 1420HH, *n*=4. IS2-S7W1 and S7W2 (3 differentiations): 1420RR, *n*=3; 1420RH, *n*=3; 1420HH, *n*=3. T- tolbutamide (100μmol/l), 2G - 2mmol/l glucose, 20G - 20mmol/l glucose. Data shown as mean and SEM.

ESM Fig 11: Insulin secretory response to increasing glucose concentration from IS1 SUR1 1420RR and 1420RH mature SC-islets

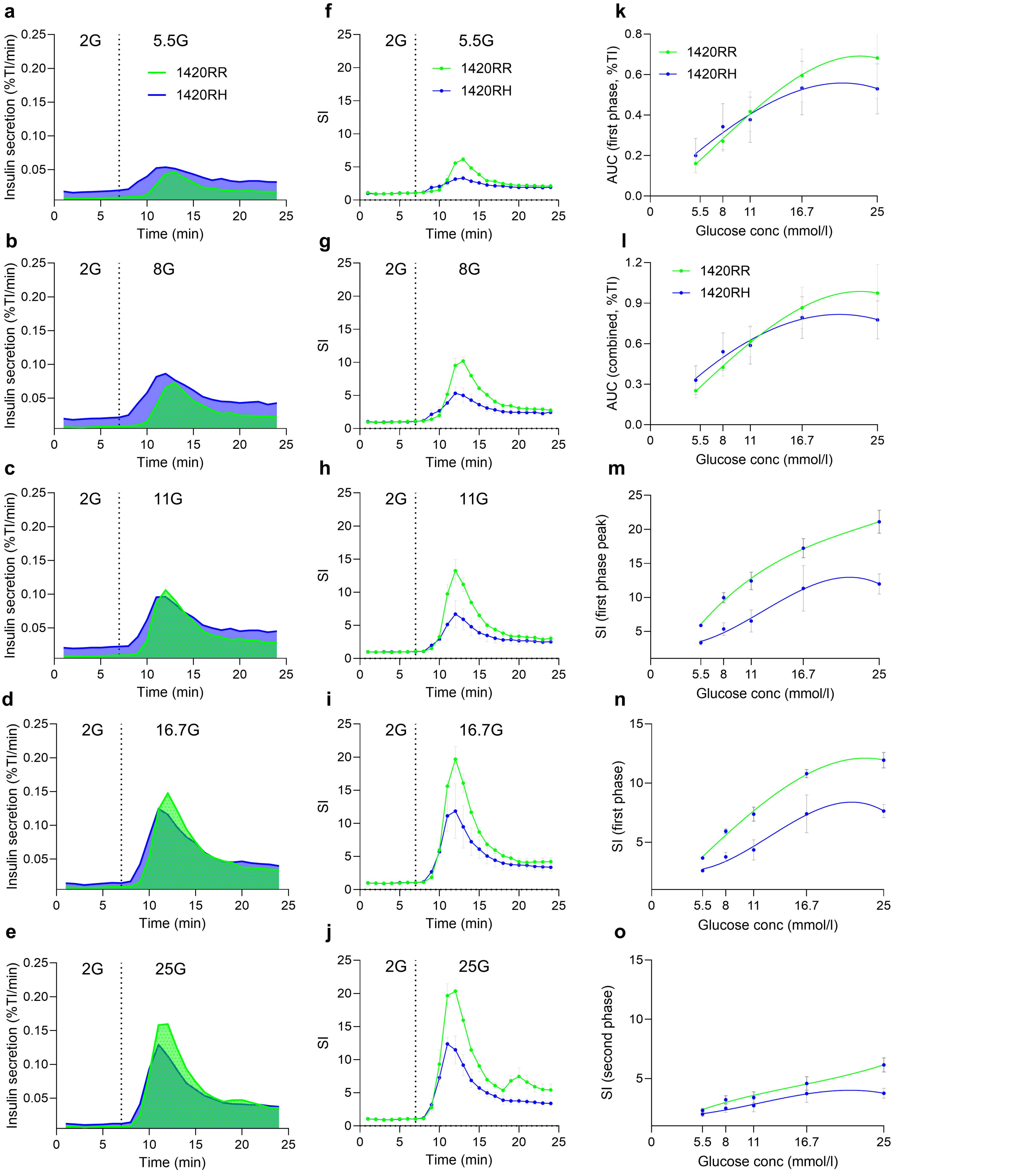

Insulin secretory response to increasing glucose concentration from IS1 SUR1 1420RR and 1420RH mature SC-islets. **a-j**. Insulin secretory response (% of total insulin) to 5.5-25mmol/l glucose (a-e) or stimulation index (f-j) from SUR1 1420RR or 1420RH mature IS1 SC-islets. **k-l**. AUC for insulin secretion during 5.5-25mmol/l glucose stimulation (l) or during first phase insulin secretion (k). **m-o**. SI during first phase (n), second phase (o) or peak (m) insulin secretion in response to 5.5 to 25mmol/l glucose. Data from 3 independent differentiations, *n*=3 for both 1420RR and 1420RH. Curves were fitted using non-linear regression (3-factor polynomial). Data shown as mean and SEM.

ESM Fig. 12: Single-cell RNA-seq to explore transcriptomic differences in 1420RH and 1420HH SC-islets.

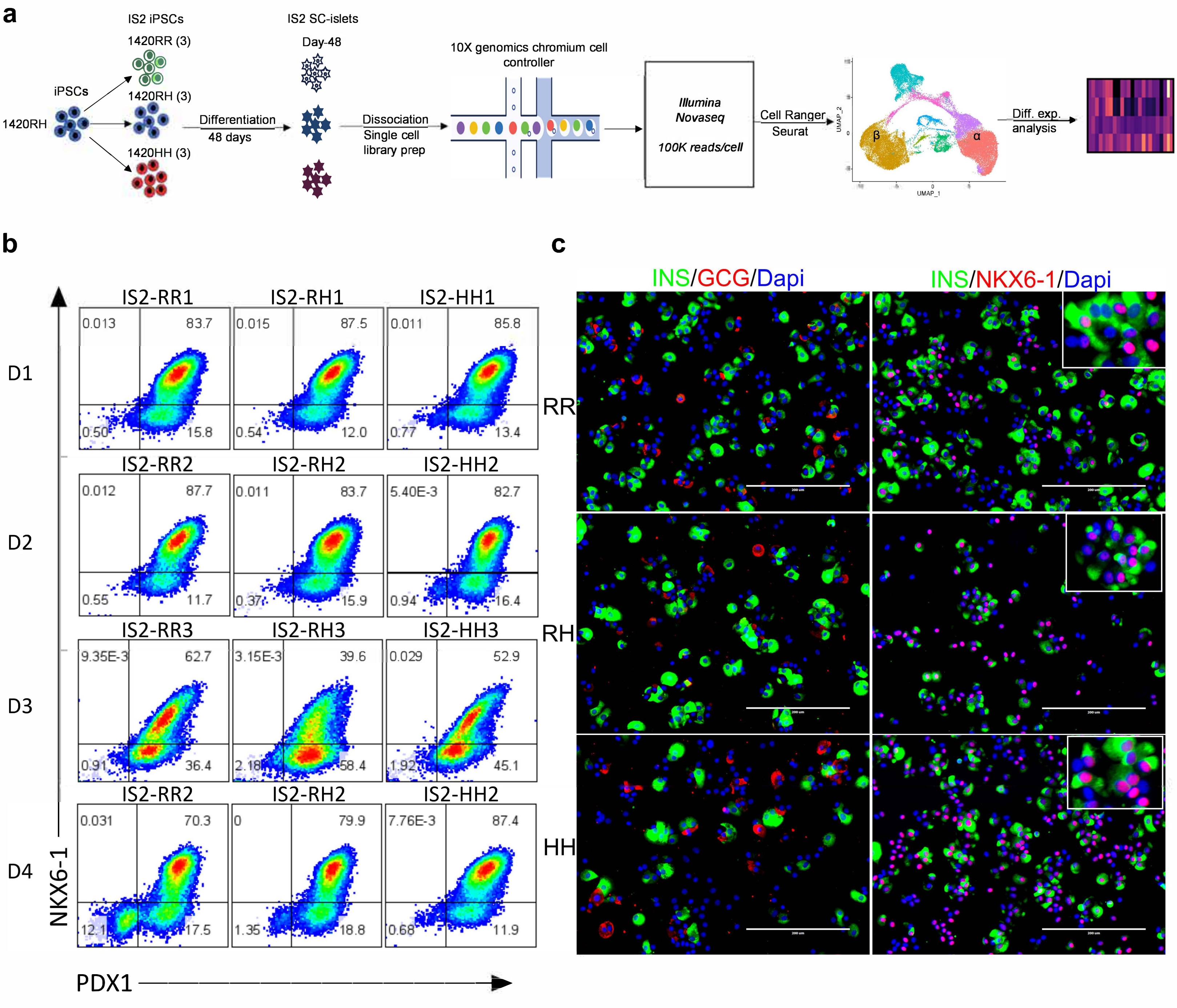

Single-cell RNA-seq to explore transcriptomic differences in 1420RH and 1420HH SC-islets. a. Outline of single cell sequencing experiment. 35,593 cells sourced from S7W4 (day 48) IS2 SC-islets generated from 4 independent differentiations were used for single cell analysis (IS2 differentiations 1 - 4, *n*=9 isogenic cell lines, 3 cell lines for each genotype). Three cell lines, one for each genotype, were used in two independent differentiations (D2 and D4). **b.** Differentiation efficiency was monitored by flow cytometry staining of PP markers PDX1 and NKX6-1 on S4D4 (day 10) and **c.** Generation of SC-beta and SC-alpha cells were confirmed by immunofluorescence staining of S7W2 SC-islets (day 34) for INS, GCG and NKX6-1 (representative images are shown).

ESM Fig. 13: Cell type recovery and polyhormonal cells after single-cell RNA-seq

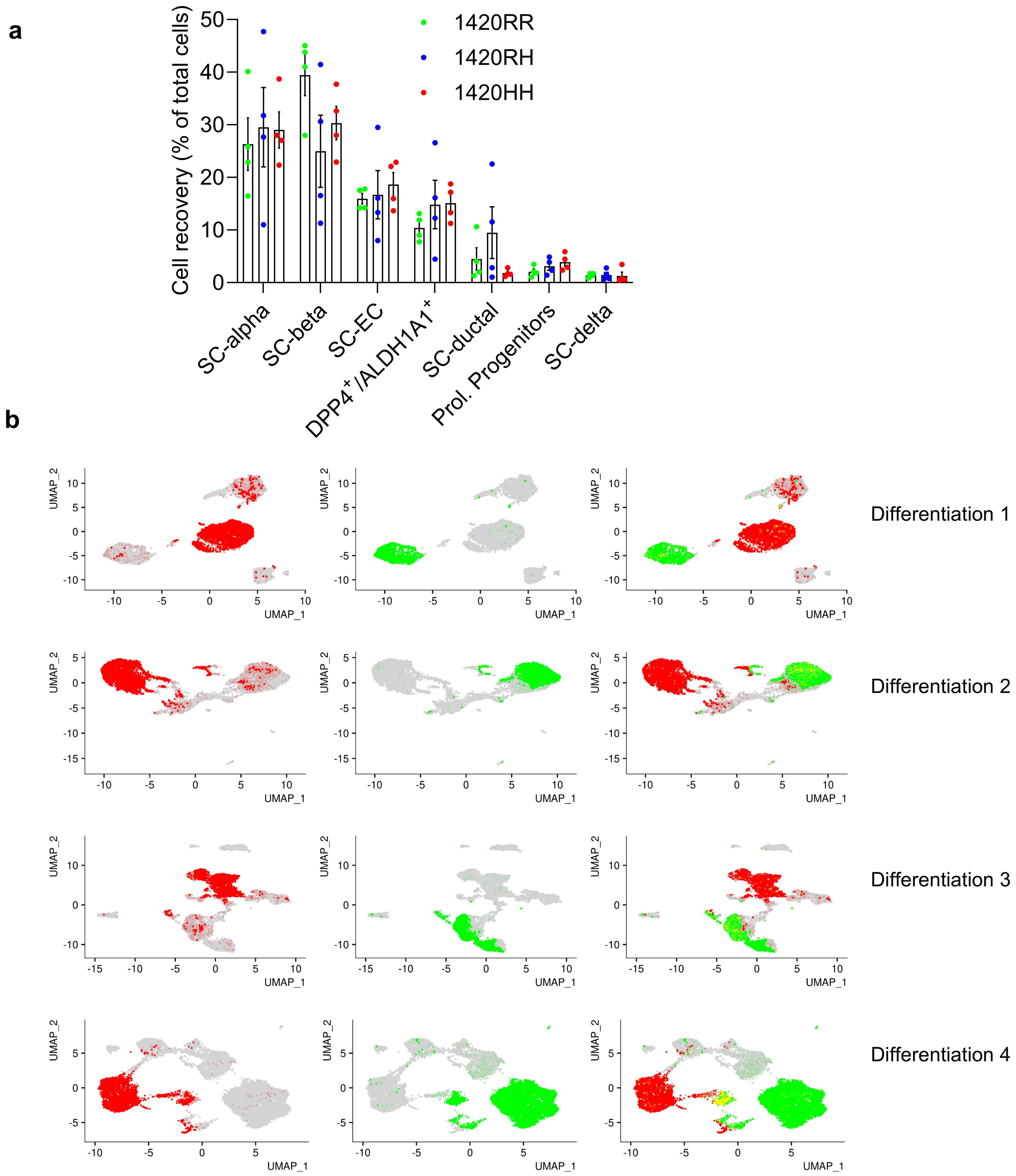

Cell type recovery and polyhormonal cells after single-cell RNA-seq. a. Proportion of cell types recovered for each genotype after single-cell sequencing from each differentiation (IS2 cell lines: differentiations 1-4, *n*=9 cell lines, 3 cell lines for each genotype. Same cell lines were used for differentiation 2 and differentiation 4 as indicated in ESM Fig. 12) **b**. UMAP projection of cells from each differentiation and identification of cells expressing *INS* (red) and *GCG* (green) and both *INS* and *GCG* (yellow).

ESM Fig. 14: Real-time PCR assessment of select dysregulated genes identififed by single cell sequencing using bulk-RNA

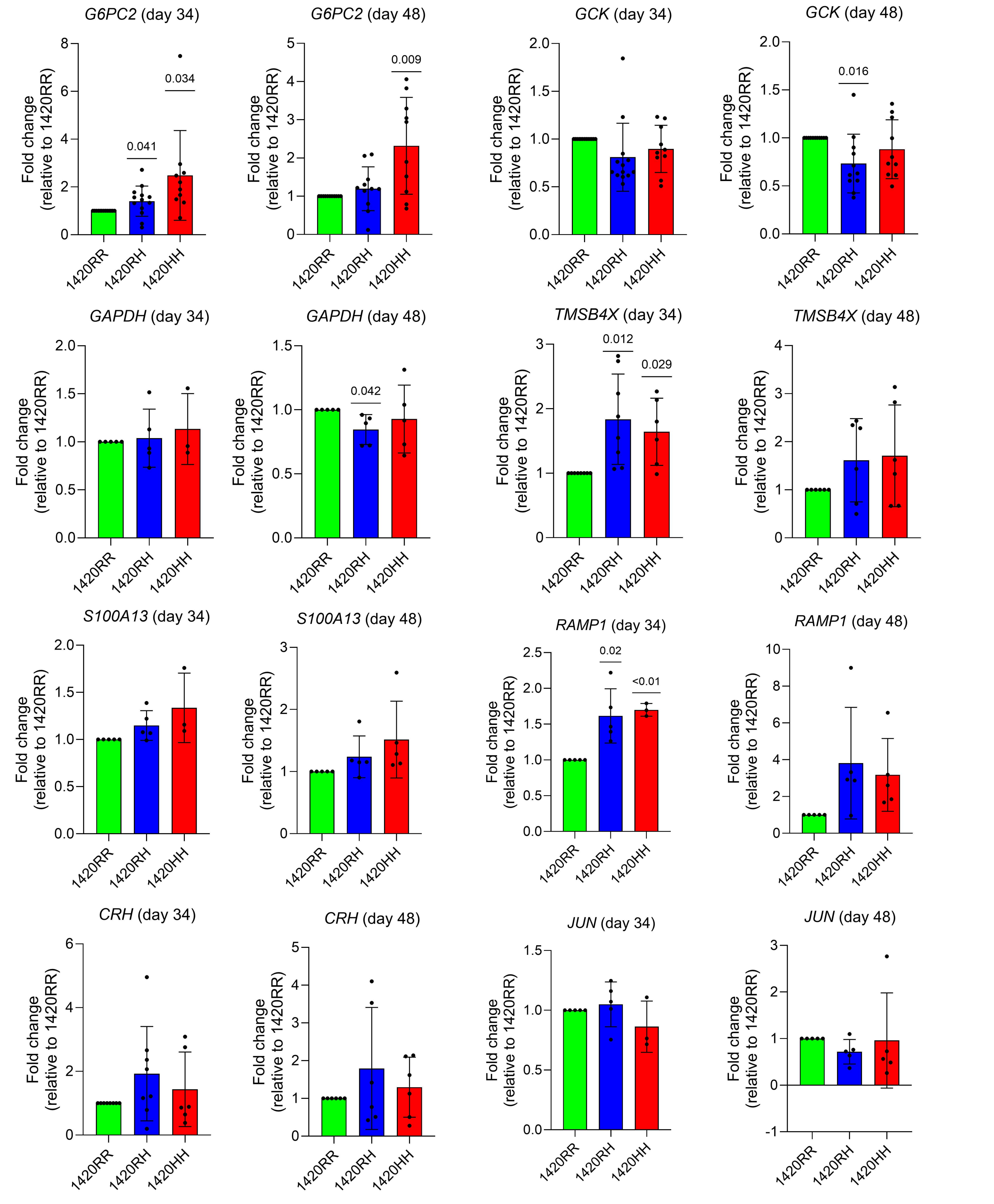

Real-time PCR assessment of select dysregulated genes identified by single-cell RNA-seq using bulk-RNA: Expression of select genes, identified to be dysregulated in 1420RH and 1420HH SC-beta and SC-alpha cells, were assessed by real-time PCR using bulk RNA from S7W2 (day 34) and S7W4 (day 48) mature IS1 and IS2 SC-islets. Data is shown as fold change in gene expression in both IS1 and IS2 1420RH and 1420HH SC-islets relative to 1420RR SC-islets from each differentiation (black dots) and represented as mean fold change ± SD. For differentiation with multiple cell line/genotype, mean fold change in gene expression in 1420RH and 1420HH (relative to 1420RR) in the respective differentiation was calculated and used for analysis and graphical presentation. Data were compared using a one sample t-test and only $p < 0.05$ is shown in the figure. **Day 34:** *G6PC2* and *GCK*; 1420RR, *n*=13, 1420RH, *n*=13 and 1420HH, *n*=10, *CRH* and *TMSB4X*; 1420RR, *n*=8, 1420RH, *n*=8 and 1420HH, *n*=6, for all other genes (only IS1); 1420RR, *n*=5, 1420RH, *n*=5 and 1420HH, *n*=3. **Day 48**: *G6PC2*, and *GCK*; 1420RR, *n*=11, 1420RH, *n*=11 and 1420HH, *n*=10, *CRH* and *TMSB4X*; *n*=6 for all three genotypes; for all other genes; *n*=5 for all three genotypes. "*n*" represents the number of independent differentiations. All real-time PCR were performed in triplicates. Cell lines included in each differentiation is shown in ESM table 2.

Appendix 1: CONSIDER checklist

Governance

1. **Describe partnership agreements between the research institution and Indigenous-governing organization for the research, (e.g., Informal agreements through to MOU (Memorandum of Understanding) or MOA (Memorandum of Agreement)).**

   Our relationship with the Tribe began in the year 1965, at a time when there was no Tribal Council subcommittee or process in place for approval of medical research protocols. Therefore, the longitudinal protocol was presented and approved directly by the Tribal Council. Many of our clinical staff members, and all recruiters, were members of the Tribal community. Annual Written Reports were provided to the Community which detailed our research progress. In more recent years, Tribal Health Committees have been created and newer protocols obtained written Tribal resolution approvals. Every 6 months we meet with Tribal Health Committees to discuss research topics and pre-submission manuscripts.

2. **Describe accountability and review mechanisms within the partnership agreement that addresses harm minimization.**

   The National Institutes of Health has recently formed the Tribal Health Research Office that reviews NIH protocols with significant Indigenous participation to identify and address areas of potential harm. In addition, protocols also require Tribal approval, and any potential harm can be identified during the review. Our regularly occurring meetings with the Tribe have also led to a better understanding of areas of societal harm. For example, to minimize stigmatization we no longer identify the Tribal community in public presentations or publications. We have also stopped using the term “mutation” in the context of human DNA variations since this word can be interpreted negatively in non-scientific settings.

3. **Specify how the research partnership agreement includes protection of Indigenous intellectual property and knowledge arising from the research, including financial and intellectual benefits generated (e.g., development of traditional medicines for commercial purposes or supporting the Indigenous community to develop commercialization proposals generated from the research).**

   A new MOU is currently pending between the US government and the tribe that will specifically address financial and intellectual benefits. In the current paper, which was reviewed by the Tribal Research and Health Committees, no patent, drug discovery or financial benefit is expected.

__Prioritization__

4. **Explain how the research aims emerged from priorities identified by either Indigenous stakeholder, governing bodies, funders, non-government organization(s), stakeholders, consumers, and empirical evidence**

   Diabetes and complications from diabetes are a major concern with the tribe. The research aims to identify the mechanism whereby a community enriched variant results in hyperinsulinaemic hypoglycaemia during infancy and doubles the risk for type 2 diabetes, and test known drugs to determine if they benefit community members who are variant carriers.

__Relationships (Indigenous stakeholders/participants and Research team)__

5. **Specify measures that adhere and honour Indigenous ethical guidelines, processes, and approvals for all relevant Indigenous stakeholders, recognizing that multiple Indigenous partners may be involved, e.g., Indigenous ethics committee approval, regional/national ethics approval processes.**

Our studies and papers are presented to tribal leadership and their trusted science advisors for input on ethical and scientific issues. The community has requested that they not be named in publications, and that data not be placed in publicly available repositories, and we have honoured these requests.

6. **Report how Indigenous stakeholders were involved in the research processes (i.e., research design, funding, implementation, analysis, dissemination/recruitment).**

   When the R1420H variant was identified, information on the risk of homozygous inheritance was presented to the Research Review Committee of the Indigenous Community, medical staff in the community and medical staff in local urban hospitals that serve Indigenous Community members. Letters that we sent to research participants who were carriers were first shown to tribal representatives for comment and approval. These letters encouraged participants to call our clinic if interested in learning of our results, and individuals who contacted us were offered free CLIA certified testing and counselling. The tribe was interested in the follow up studies of this variant and the current work was reviewed by the Tribal Research and Review Committee and Tribal Health and Social Standing Committee.

7. **Describe the expertise of the research team in Indigenous health and research.**

   The research team has been conducting studies in partnership with Indigenous Communities for more than 30 years. Most of our recruiters were members of the same community where our research was conducted. In addition, most of our clinical staff is Indigenous as are many members of the basic science research team.

Methodologies

8. **Describe the methodological approach of the research including a rationale of methods used and implication for Indigenous stakeholders, e.g., privacy and confidentiality (individual and collective)**

   The Tribal name and Community are not mentioned in the publication. All data are de-identified and are exempt from putting into public databases.

9. **Describe how the research methodology incorporated consideration of the physical, social, economic and cultural environment of the participants and prospective participants. (e.g., impacts of colonization, racism, and social justice). As well as Indigenous worldviews.**

   Given that some Indigenous community members feel they are being used for research for the purpose of helping wealthy Caucasian communities, it was vital that our study sought to identify known drugs that may help individuals with early onset type 2 diabetes and infants with hyperinsulinaemic hypoglycaemia *due to a specific DNA variation that is highly enriched in a specific Indigenous Community*. To account for any genomic or epigenomic differences, all studies were done with cells from consented Indigenous participants from the same community. The research presented in the manuscript was only possible because of the continuous engagement with the Tribal Community. The identification of this variant, the effect of the variant on Indigenous health and the induced pluripotent stem cell-based modelling of this variant (this manuscript) occurred over the span of many years with repeated presentations and approvals by the Tribal Health Committees.

Participation

10. **Specify how individual and collective consent was sought to conduct future analysis on collected samples and data (e.g., additional secondary analyses; third parties accessing samples (genetic, tissue, blood) for further analyses).**

    The consent forms clearly stated that samples would be continued to be used for future studies and that data could be analysed for diabetes-related studies. Additional secondary analyses and sharing of data with third parties requires review by the relevant tribal committees. The consent form was also reviewed by the Tribal Research and Review Committee and the Tribal Health and Social Standing Committee.

11. **Described how the resource demands (current and future) placed on Indigenous participants and communities involved in the research were identified and agreed upon including any resourcing for participation, knowledge, and expertise**

    The NIH IRB approves monetary amounts provided for participation in NIH protocols; the amount given for each procedure is based on the PI proposed number of "inconvenience units," and the monetary value of each unit. These IRB approved amounts and time commitments are clearly delineated in protocols presented to the tribe. However, this is indeed an important issue and the topic of appropriate compensation for Tribal members volunteering for studies (fair compensation versus monetary inducement) was broadly discussed with us at a recent Tribal Council meeting.

12. **Specify how biological tissue and other samples including data were stored, explaining the processes of removal from traditional lands, if done, and of disposal.**

All biologic materials are securely stored and de-identified in our branch which is not on tribal land. The tribe has an inventory of our samples. Samples are not disposed.

Capacity

13. **Explain how the research supported the development and maintenance of Indigenous research capacity (e.g., specific funding of Indigenous researchers).**

Our branch has trained more than 100 indigenous students of which **27 of our Indigenous trainees have subsequently obtained either a MD or PhD.** Some of these individuals have returned to their community as physicians, while many are actively involved in Advancing Indigenous People in STEM (AISES). These trainees also help our non-Indigenous staff understand cultural differences and the sanctity of human biospecimens. Additionally, the research work in this manuscript was presented and discussed during the Tribal Health Committee meetings allowing for bidirectional learning.

14. **Discuss how the research team undertook professional development opportunities to develop the capacity to partner with Indigenous stakeholders?**

Our group currently has more than 60 years of experience in working with Indigenous stakeholders. Currently, a key area of partnership involves the use of DNA in medical research. Members of our research team have attended a meeting on Precision Medicine Research with American Indian and Alaska Native Communities, where tribal representatives from Alaska and the Pacific Northwest had round table discussions with medical researchers and very recently participated in the 2025 Voices of the Diné: Bridging Science, Culture, and Community in Genetic Research Summit.

Analysis and interpretation

15. **Specify how the research analysis and reporting supported critical inquiry and a strength-based approach that was inclusive of Indigenous values.**

We understand and respect the sanctity of DNA among Indigenous people. We also respect the wishes of the community and the concerns of Indigenous Communities about certain lines of investigation including migration patterns and tribal relatedness.

A key aspect of this study is that we are studying the effect and treatment of a DNA variant in the same genomic background as people who are affected by this variant. Given that some of the donor's methylation patterns are also preserved in islets derived from iPSCs, it is possible that we are also capturing the epigenomic background of the community affected by this variant. Environment, chronic stress, and exposure to various socio-economic factors have all been shown to cause epigenetic changes. Therefore, our study may be capturing important information on therapeutic options that best serve not only people who have similar DNA but also share similar life exposures.

Dissemination

16. **Describe the dissemination of the research findings to relevant Indigenous governing bodies and peoples.**

For decades we have provided written reports to the tribe about all our studies and publications. More recently we meet with tribal committees every 6 months to provide updates. The results of the current study were orally presented to the Tribal Research and Review Committee. The manuscript was reviewed by the Tribal Research and Review Committee and the Tribal Health and Social Standing Committee.

17. **Discuss the process for knowledge translation and implementation to support Indigenous advancement (e.g., research capacity, policy, investment).**

NIDDK has had a program to recruit Indigenous American and Native Alaskan undergraduate students to work in our labs over the summer. We also partner with universities such as Northern Arizona University which have a higher percentage of Indigenous undergraduates to hire recent graduates to obtain positions in our clinical and research laboratories. This has resulted in our branch training more than 100 Indigenous students of which 27 of our Indigenous trainees have subsequently obtained either a MD or PhD.

This study represents an excellent example of how genetic research in an Indigenous people can directly help their community. This variant is highly enriched in this community, and therefore homozygous inheritance is a reality. Since different variations in *ABCC8* do not respond to diazoxide, it is important for community health care providers to know that infants homozygous for this variation are likely to be responsive to diazoxide in case of severe hypoglycaemia. The Tribal Health Committees have also been made aware of this during discussions of this manuscript.
